# At least it is a dry cold: the global distribution of freeze–thaw and drought stress and the traits that may impart poly-tolerance in conifers

**DOI:** 10.1093/treephys/tpac102

**Published:** 2022-09-12

**Authors:** Katherine A McCulloh, Steven P Augustine, Alex Goke, Rachel Jordan, Christopher P Krieg, Kimberly O’Keefe, Duncan D Smith

**Affiliations:** Department of Botany, University of Wisconsin-Madison, Madison, WI, 53706, USA; Department of Botany, University of Wisconsin-Madison, Madison, WI, 53706, USA; Department of Botany, University of Wisconsin-Madison, Madison, WI, 53706, USA; Department of Botany, University of Wisconsin-Madison, Madison, WI, 53706, USA; Department of Botany, University of Wisconsin-Madison, Madison, WI, 53706, USA; Department of Biological Sciences, Saint Edward’s University, Austin, TX 78704, USA; Department of Botany, University of Wisconsin-Madison, Madison, WI, 53706, USA

**Keywords:** abiotic stress, conifers, distributions, drought, freeze-thaw, poly-tolerance

## Abstract

Conifers inhabit some of the most challenging landscapes where multiple abiotic stressors (e.g., aridity, freezing temperatures) often co-occur. Physiological tolerance to multiple stressors (‘poly-tolerance’) is thought to be rare because exposure to one stress generally limits responses to another through functional trade-offs. However, the capacity to exhibit poly-tolerance may be greater when combined abiotic stressors have similar physiological impacts, such as the disruption of hydraulic function imposed by drought or freezing. Here, we reviewed empirical data in light of theoretical expectations for conifer adaptations to drought and freeze–thaw cycles with particular attention to hydraulic traits of the stem and leaf. Additionally, we examined the commonality and spatial distribution of poly-stress along indices of these combined stressors. We found that locations with the highest values of our poly-stress index (PSi) are characterized by moderate drought and moderate freeze–thaw, and most of the global conifer distribution occupies areas of moderate poly-stress. Among traits examined, we found diverse responses to the stressors. Turgor loss point did not correlate with freeze–thaw or drought stress individually, but did with the PSi, albeit inverse to what was hypothesized. Leaf mass per area was more strongly linked with drought stress than the poly-stress and not at all with freeze–thaw stress. In stems, the water potential causing 50% loss of hydraulic conductivity became more negative with increasing drought stress and poly-stress but did not correlate with freeze–thaw stress. For these traits, we identified a striking lack of coverage for substantial portions of species ranges, particularly at the upper boundaries of their respective PSis, demonstrating a critical gap in our understanding of trait prevalence and plasticity along these stress gradients. Future research should investigate traits that confer tolerance to both freeze–thaw and drought stress in a wide range of species across broad geographic scales.

## Teaser

- Majority of conifer extent subjected to moderate poly-stress.
- Traits that correlated with drought stress also correlated with poly-stress.
- Paucity of trait data reflects a need for range-wide sampling.

## Introduction

Globally, coniferous forests experience harsh and stressful environmental conditions throughout most of the year. Plants in these ecosystems may be exposed to a range of weather extremes, including freezing temperatures in the winter, high air temperatures in the summer and low water availability seasonally. Transitions between frozen and thawed states (i.e., ‘freeze–thaw’ cycles) and/or periods of drought can impair physiological functioning in a variety of ways, including photosynthetic decline, hydraulic dysfunction and mortality (Huner et al. 1998, 2003, Allen and Ort 2001, Ivanov et al. 2001, Pittermann and Sperry 2003, 2006, Mayr et al. 2006, Choat et al. 2018, Blackman et al. 2019). Despite recent progress in characterizing stress mitigation and tolerance mechanisms to single stressors (e.g., drought, Choat et al. 2018; freeze–thaw, Guy 2003, Zanne et al. 2014), we currently lack a comprehensive understanding of the mechanisms by which evergreen conifers mitigate multiple (but not necessarily concurring) physiological stresses (i.e., ‘poly-stress’). In the context of a changing climate, it is particularly important that we understand how plants tolerate stressors that currently exhibit strong selective pressure, such as drought and freeze–thaw cycles, in order to improve our predictions of how forest compositions may shift in the future.

Both drought and freeze–thaw cycles are expected to become more frequent in many coniferous forest regions due to rising air temperatures and shifting precipitation regimes associated with global climate change (Henry 2008, IPCC 2019). Global warming, which is greatest at higher latitudes, can lead to mid-winter warming events (Williams et al. 2015), increase the occurrence of rain relative to snow (Knowles et al. 2006), decrease total snow accumulation (Mote et al. 2005) and result in earlier spring snowmelt (Clow 2010). Increased variability in winter temperatures and reduced insulation from temperature fluctuations associated with lower snow cover can increase the frequency of freezing and thawing experienced by plants (Henry 2008). These conditions can also have cascading consequences throughout the growing season as reduced snow cover and, consequently, diminished and less prolonged snowmelt limit available water for plants throughout the growing season (Hu et al. 2010, Williams et al. 2015). When combined with the elevated summer air temperatures and reduced summer precipitation predicted for some of these regions, many trees will likely experience population declines associated with these combined stresses (Mayr et al. 2006, Earles et al. 2018).

Freeze–thaw cycles and drought impose similar impacts on leaves and stems. In leaves, both freezing temperatures and drought can hinder photosynthetic capacity and lead to excess absorbed light energy that causes photoinhibitory damage and leaf death (Demmig-Adams and Adams 2006). In stems, both freeze–thaw cycles and drought disrupt the hydraulic network by inducing or propagating embolisms throughout the xylem (Tyree and Sperry 1989). Although the mechanism of formation differs, embolisms caused by both freeze-thaw events and drought reduce whole plant hydraulic conductance, which can limit plant functioning and growth (Kreyling 2010, Anderegg et al. 2016). Given that these stresses create similar dysfunction in plants, as well as the observations that evergreen conifers often experience freeze–thaw cycles and drought simultaneously in some habitats (Mayr et al. 2006) and that these stresses are likely to have compounding effects (Charrier et al. 2021), the survival and distribution of these taxa likely depends on a tolerance to both stressors (i.e., ‘poly-tolerance’). However, some studies have noted a distinct trade-off between drought and cold/freezing tolerance in woody taxa mediated by reduced wood density in frost-tolerant species (e.g., Laanisto and Niinemets 2015, Rueda et al. 2017), suggesting divergent trait coordination that promotes either freeze–thaw or drought tolerance across large geographic scales. Though some studies have systematically examined organ-level traits that could promote tolerance to multiple abiotic stressors across a wide range of species (e.g., Hallik et al. 2009, Stahl et al. 2013, Rueda et al. 2017), no study has quantified the degree of geographic overlap between freeze–thaw and drought stress to identify key areas where poly-tolerance to these stressors would be most adaptive. Additionally, we do not yet have a comprehensive understanding of what traits confer both freeze–thaw and drought tolerance, how widespread the coordination of these traits is across species or how these traits might influence the survival and distribution of conifers worldwide.

In this review we aim to (i) examine indices of the worldwide spatial distribution of drought, freeze–thaw cycles (hereafter, ‘FT cycles’) and their combination, and (ii) identify traits that may impart tolerance to drought and FT cycles, and explore to what extent these traits overlap in evergreen conifers. In particular, we focused this review on the physiological, morphological and anatomical traits of conifer leaves and stems. Although roots are also susceptible to freezing and drought stresses, comparatively little research has investigated the topic (but see Ambroise et al. 2020 for a review on frost resistance in crop roots). This review is not intended to comprehensively examine all aspects of winter or drought ecophysiology (but see Chang et al. 2021, Choat et al. 2018, McDowell et al. 2008, Sakai and Larcher 2012). Other recent reviews have covered topics such as extreme winter weather events (Casson et al. 2019); winter climate change impacts on plant species composition, ranges and phenology (Kreyling 2010); and the impacts of severe drought on global forest mortality (Allen et al. 2010, Anderegg et al. 2013, Clark et al. 2016). By identifying the global occurrence of FT and drought, as well as relevant traits that may enhance tolerance to these combined stressors, we aim to identify promising research avenues that will enhance predictions of conifer function, abundance and distributions in a changing climate.

## Assessing the worldwide prevalence of drought stress, FT stress and their combination

**Figure 1.**
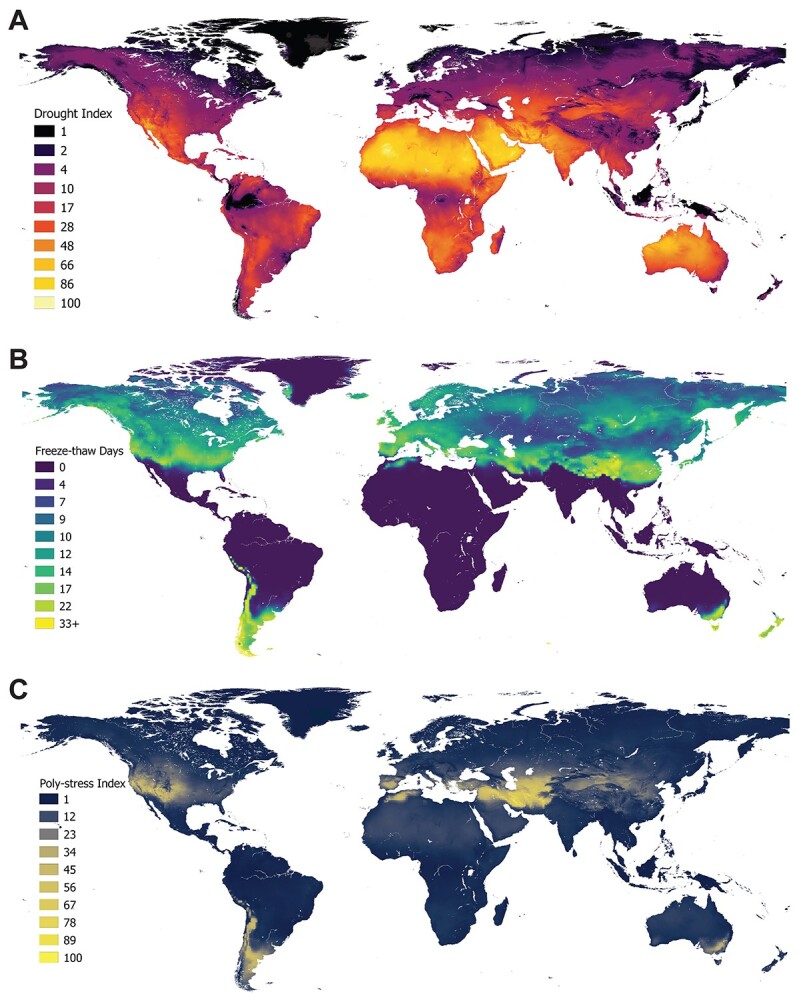
Spatial distribution of (A) annual average Di, (B) number of freeze–thaw days and (C)a PSi based on their spatial overlap. Indices (A, C) range from 1 to 100 in order of increasing stress. Freeze–thaw days are shown in (B) for interpretability of common units (days), see Supplemental Methods for details about the conversion to the freeze–thaw index (FTi) used in other figures. Details of both indices (A, C) are described in the methods. Color thresholds were determined to maximize contrast.

Drought and freezing temperatures profoundly influence the global distribution of plant groups (Stuart et al. 2007, Engelbrecht et al. 2007, Normand et al. 2009, Stahl et al. 2014). The distribution and trait evolution of evergreen woody angiosperms can be sharply defined by exposure to freezing temperatures (Zanne et al. 2018), while maximum temperature and precipitation are more strongly associated with tolerance traits and distributions in conifers (Rueda et al. 2017). This suggests a role for overlapping stressors as a primary driver of conifer occurrence as opposed to temperature or precipitation singly. Furthermore, the impacts of minimum annual temperatures on conifer traits and distributions are likely small compared with the impacts of the FT cycles that occur in cooler climates. Freezing temperatures alone can reduce photosynthetic capacity (Huner et al. 1998, 2003, Ivanov et al. 2001) and increase the hydraulic resistance in soil (Tranquillini 1976), while FT cycles can cause severe hydraulic dysfunction in xylem via embolisms (Hammel 1967, Sucoff 1969). Cold hardiness, studied extensively in conifers, often varies by genotype and is correlated with local minimum temperatures (e.g., Sebastian-Azcona et al. 2018), although foliage is commonly hardened to temperatures far beyond those minimums (Sakai 1960, Strimbeck et al. 2007, Wisniewski et al. 2018). However, there is no broad-scale relationship between minimum temperature and FT events (Figure S1 available as Supplementary data at *Tree Physiology* Online). As such, common ranking systems for cold tolerance or cold hardiness do not show any clear relationship with FT events (Figure 1 available as Supplementary data at *Tree Physiology* Online), thus providing an opportunity to investigate the spatial distribution and impact of FT cycles on plant function and ecology.

**Figure 2.**
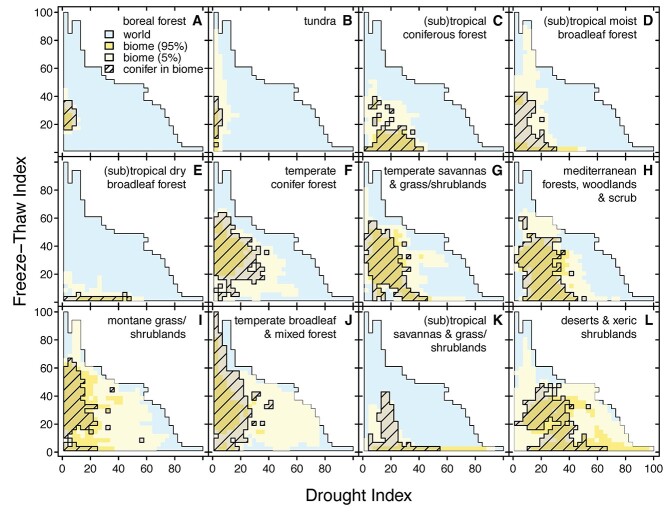
The distribution of 12 major biomes in FTi–Di space, relative to the occurrence of all global landmass (excluding Antarctica - blue; ``world'') are shown in shades of yellow (“biome”). The darker shade indicates the bulk of common FTi–Di combinations (“biome 95%”), while the pale shade shows the remaining, rare combinations (“biome 5%”). Hatched regions indicate the reported occurrences of conifer species within each biome. Drought and freeze–thaw indices range from 1 to 100 in order of increasing stress. Each pixel is 3 × 3 index units. For every conifer species, we included it in a biome only if at least 5% or 10 observations were recorded in that biome.

To visualize the global patterns of drought, FT cycles and their combination, we used available global climate data to develop spatial indices of drought stress (Di, based on soil water content and vapor pressure deficit (VPD); comparison with other water availability metrics shown in Figure S2 available as Supplementary data at *Tree Physiology* Online), FT stress (FTi, based on daily maximum and minimum temperatures) and poly-stress index (PSi, based on the product of the individual stresses; see Supplemental Materials for detailed methods). Our PSi shows bimodal latitudinal peaks at ca. 34.8^o^S and 32.8^o^N (Figure 1, and Figure S3 available as Supplementary data at *Tree Physiology* Online), and the distribution of conifers overlaps considerably with areas of land that experience moderate levels of FT and drought stresses (i.e., index values of 30–40; Figure 2). Regions of moderate poly-stress that have high conifer abundance and diversity include the western USA, southern Argentina and Chile, northern Morocco, the Iberian Peninsula and the northern extents of central Asia (Sundaram et al. 2019). Locations that have more extreme values of the PSi (i.e., high index values), including Iran and southeastern Australia, are rare globally (Figure S4 available as Supplementary data at *Tree Physiology* Online) and are dominated by angiosperms, which are cold deciduous in Iran but evergreen in Australia. The PSi does not indicate high levels of poly-stress in the majority of the vast boreal forests of the northern hemisphere (Figure 1C), which are instead characterized by a high number of FT days but low drought stress (Figure 1A and B). It is worth noting, though, that some regions of the boreal forest do experience some drought stress, particularly in British Columbia, Canada, which has recently experienced severe heatwaves and droughts that have exacerbated widespread wildfires. For the majority of the boreal forests that experience little water stress, the main climatic driver of this biome type is likely associated with the stresses of repeated freezing and thawing, along with the extreme cold. At extreme degrees of drought, conifers are generally replaced by plants (often angiosperms) that exhibit succulence and CAM photosynthesis, or the rapid growth of desert annuals (however, see Larter et al. 2015, 2017), while at extreme FT stress, conifers are replaced by deciduous angiosperms. In locations with relatively extreme poly-stress, the vegetation type tends to be desert and xeric shrubland without conifers (Figure 2L).

Importantly, the data used to quantify the distributions of drought, FT and poly-stress (e.g., Figure 1) are from the recent past (see Supplemental Materials for details). As the global climate changes, shifting precipitation patterns will reduce soil moisture availability in some areas (Dai et al. 2018) and higher temperatures will increase the VPD broadly (Yuan et al. 2019). Furthermore, variations in the extent and location of polar vortices, along with milder winter temperatures in the arctic, could increase the number of FT cycles in regions of the northern hemisphere (J. Zhang et al. 2016, Matsumura et al. 2021). Thus, the spatial area experiencing multiple stresses should increase in the future, as should the intensities of poly-stress.

## Traits and stress tolerance

**Figure 3.**
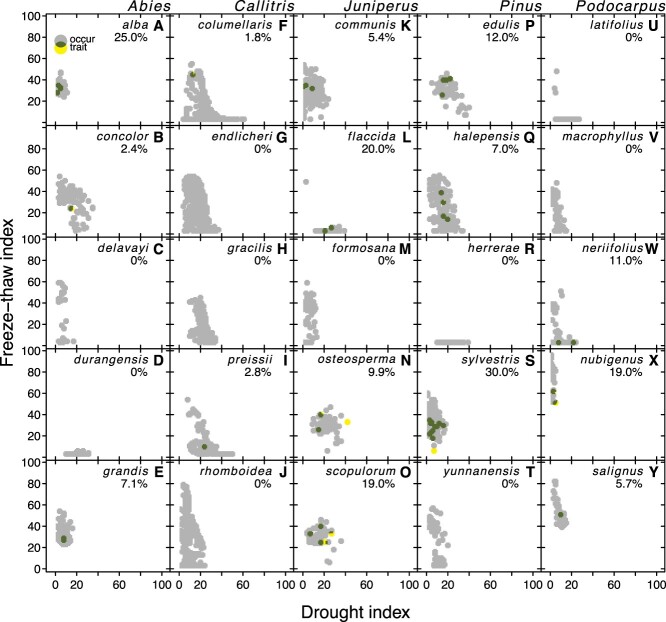
The availability of occurrence data (grey; “occur”), trait data (yellow; “trait”) and their overlap (dark green) in FTi-Di space for five representative species in five representative genera. Each data point was rounded to the nearest FT and drought index and then buffered by a radius of two index units. The approximate completeness of trait data that is available relative to the environmental space that each species occupies was calculated as the area of yellow and dark green pixels divided by the area of yellow, dark green and grey pixels, and expressed as a percentage in the top right of each panel.

The apparent biome shifts away from conifers at the extreme abiotic stress levels in Figure 2 likely represent the physiological limits of conifers. However, which specific traits limit individual species within this broader distribution remains unclear. Figure 3 shows the occurrence records of multiple species from five genera along the axes of the FT and drought stress indices, illustrating that these taxa vary considerably in their distribution within our poly-stress space. Some species are absent in areas of drought stress but are present in areas of high FT stress (e.g., *Podocarpus nubigenus*, Figure 3W), while others show the reciprocal pattern (e.g., *Pinus herrerae*, Figure 3R), suggesting a trade-off. Indeed, we found a significant negative relationship between species’ median positions on the drought and FT stress index (R^2^ = 0.19, *P* < 0.001; data not shown). However, some species occur in areas subject to both stressors (e.g., *Pinus halepensis*, Figure 3Q).

In the next sections, we discuss specific traits that could impart tolerance to drought, FT cycles and their combination in conifer leaves and stems, thus influencing their geographical distribution and position on the Di, FTi and PSi. Comparative studies on interspecific hydraulic traits have shown significant relationships between these traits and either drought or FT cycles (Maherali et al. 2004, Pittermann and Sperry 2006, Bartlett et al. 2012, Choat et al. 2012, Larter et al. 2017, Rosas et al. 2019, Sanchez-Martinez et al. 2020, Rosas et al. 2021), which may provide insight in to mechanisms facilitating the tolerance of particular conifer species to both stresses. We test how these traits relate to drought and FT stresses using data compiled from databases and our own literature search (see Supplemental Materials). Relationships were analyzed using linear regressions. We checked the appropriateness of a linear model with a Shapiro normality test. All models that included leaf mass per area (LMA) failed to meet the proper assumptions and data were log-transformed.

### Traits that impart tolerance in leaves

**Figure 4.**
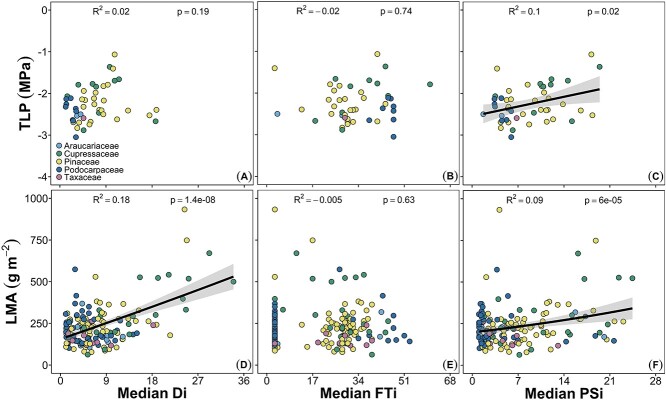
Linear regression of mean species’ turgor loss point (TLP; *n* = 52) and LMA (*n* = 202) on median species’ occurrence on the drought (Di, A, D), freeze–thaw (FTi, B, E) and poly-stress (PSi, C, F) indices. Regression lines and 95% confidence intervals are presented where significant. The LMA data were not normally distributed and were log transformed. The regressions present in these panels are logarithmic. Each dot represents one species in the families indicated.

Among the most well-established characteristics associated with tolerating drought stress are those that maintain turgor pressure in living cells. Positive turgor is crucial for proper metabolic functioning within cells. Turgor can be maintained under water deficits by lowering the turgor loss point (TLP), which can occur in multiple ways. First, the TLP can be lowered through a reduction in the cell’s osmotic potential, which limits water loss. Lower TLPs associated with reductions in osmotic potentials have been studied extensively, and there is considerable evidence that across biomes, more arid-adapted plants exhibit lower TLP values due to higher concentrations of osmotically active solutes (Bartlett et al. 2012), although the majority of data come from angiosperms. When we compared the TLP of conifers reported in the literature (see Supplemental Methods) with the species’ median Di, we found no relationship (*P* = 0.19; Figure 4A). The lack of a relationship is surprising, given previous research on the importance of TLP. However, TLP in conifers is not as well studied as in angiosperms, and our dataset lacks measurements of species living in the more severe end of the Di range that conifers occupy. Second, TLP can be lowered by decreasing the elastic modulus of cell walls, which allows the cell wall to collapse slightly as water is lost (Saito and Terashima 2004). Meinzer et al. (2014) found evidence of rapid adjustment of elastic modulus when *Juniperus monosperma* leaves were rehydrated, which resulted in a far milder TLP than the trees achieved in situ. Conversely, no adjustment was observed in the elastic modulus of *Pinus edulis* leaves in the same study, nor did Bartlett et al. (2012) find evidence of elastic modulus adjustments in response to drought across a broad range of mostly angiosperm species. This discrepancy suggests not all species adjust TLP using the elastic modulus. Third, plants can reduce TLP by increasing the water content within the apoplast relative to that in the symplast (called the ‘apoplastic water fraction’, Bartlett et al. 2012). This concentrates the solutes within the cell, reduces the osmotic potential and consequently lowers the TLP (assuming the other components of cellular water relations remain constant). Regardless of how TLP is reduced or maintained, lower TLP typically results in a greater tolerance of water stress. The TLP has also been shown to be tightly coordinated with a large number of other traits that impart drought tolerance, such as the ability to maintain leaf hydraulic conductance (Brodribb and Holbrook 2006, Nardini et al. 2012, Nardini and Luglio 2014, Johnson et al. 2018, Yao et al. 2021), which is known to be an indicator of drought tolerance in conifers (Brodribb and Cochard 2009), and stomatal conductance (Brodribb and Holbrook 2003, Li et al. 2018) at low water potentials.

Extracellular ice formation is one of the most damaging aspects of FT events. The freezing point of water within the apoplast is higher than that of the cytoplasm, and consequently, intercellular water typically freezes first. Freezing lowers the water potential of the apoplast and draws water out from the living cells, which may lead to cellular dehydration and damage to the structure and function of the plasma membrane (Guy 1990, 2003). The amount of cellular dehydration that occurs with freezing is temperature-dependent because water potential declines with the temperature of the ice, which in turn reduces the water potential of the cytoplasm (Xin and Browse 2000). Thus, plants that are able to reduce the osmotic potential of living cells might experience less damage due to extracellular ice formation (e.g., *Tsuga canadensis*; Tyree et al. 1978). Indeed, substantial evidence shows that plants acclimated to cold temperatures have lower leaf osmotic potentials and also experience less freezing damage than plants not acclimated to cold temperatures prior to freezing (Kasuga et al. 2007, Charrier et al. 2013, Ting et al. 2014, Arias et al. 2015, 2017). Although a lower osmotic potential is generally associated with lower TLP, we found no significant correlation between FTi and TLP in our dataset (*P* = 0.74; Figure 4B). One explanation for the lack of a relationship is that a lower TLP does not impart greater tolerance to higher FT stress. This may be true if a threshold exists below which reducing the TLP does not increase tolerance to FT cycles. Another explanation is that the TLP values we compiled report values that were only collected in the summer. Conifer TLP values have been shown to adjust throughout the growing season in response to drought (Meinzer et al. 2014, Johnson et al. 2018), and in response to winter (Ritchie and Shula 1984, Gross and Koch 1991, Grossnickle 1992). When apoplastic water freezes and draws water from the symplast, this concentrates the solutes and reduces the osmotic potential and likely the TLP (Mayr and Améglio 2016, Lintunen et al. 2018). The adjustment of pressure–volume curve parameters in the winter deserves far greater attention.

Adjustment of the elastic modulus may also reduce damage associated with extracellular ice formation, as more rigid cell walls can prevent cell collapse (Scholz et al. 2012, Le Gall et al. 2015, Y.J. Zhang et al. 2016). Finally, the ability to adjust the apoplastic water fraction while tissues are experiencing freezing temperatures may help plants avoid ice formation and tolerate lower temperatures (Goldstein et al. 1985, Arias et al. 2015, 2017). The extent to which this pattern holds in conifers or imparts tolerance to FT cycles (and not simply low temperatures) remains unclear (Grossnickle 1992). However, the most poly-tolerant conifers would likely exhibit the greatest ability to adjust osmotic, elastic and apoplastic properties because doing so is associated with tolerance and/or avoidance to both drought and freezing. For example, the ability to seasonally adjust the apoplastic water fraction would allow summer increases during drought stress and nongrowing season decreases to tolerate frequent FT cycles. However, there are currently no data available to test if conifers adjust in this way, as the available data are heavily biased toward summertime measurements.

Given the theoretical importance of TLP in conifer leaf functioning under drought and FT, as well as empirical support showing that TLP is a key drought tolerance trait in angiosperms (e.g., Bartlett et al. 2012), we predicted conifers would also exhibit strong correlations between TLP and PSi. As discussed above for the individual stresses, the data we compiled indicated no relationships between TLP and Di or FTi (Figure 4). However, our results indicate that the PSi weakly correlated with TLP values (R^2^ = 0.1; *P* = 0.02; Figure 4C), but in the reverse direction to what we expected, with more negative TLP associated with species inhabiting low stress regions. This relationship should be explored further, and there are several things that should be considered. First, the range of TLP values is fairly limited. It would be useful to measure species expected to exhibit very negative TLP values. Second, there is evidence that some conifers are able to adjust their TLP values very rapidly and that this shift can indicate artifactually high TLP values when not accounted for (Meinzer et al. 2014). Although this has not been explored extensively, the artifactually high TLP values have been observed in *J. monosperma*, and another study found large temporal variation in TLP values over a growing season in *Juniperus asheii*, suggesting this ability may be common in the Cupressaceae. Finally, we predict that plants adapted to high PSi values would be able to adjust their TLP values under stressful conditions. If the reported TLP values were measured during favorable seasonal conditions, they could be independent of the growing conditions reflected in the PSi value. It remains unclear what is driving the patterns between the stress indices and TLP, and further investigation into this important trait is warranted.

A morphological trait of leaves that may be associated with poly-tolerance to both drought and FT cycles is LMA. Although LMA is not explicitly a hydraulic trait, changes in this trait are associated with changes in leaf hydraulic conductance expressed on a per mass basis (Nardini et al. 2012, Simonin et al. 2012). In habitats with lower water availability, leaves tend to become thicker and/or heavier for a given area (i.e., LMA increases). Mechanistically, this change is caused by more proximal responses to drought stress, such as increases in cell wall thicknesses to withstand more negative water potentials and the need for thicker, longer-lived leaves in resource-limiting environments (Givnish 2002, Hodgson et al. 2011, Simonin et al. 2012). Although LMA generally increases across species in more arid environments, the relationship is fairly weak (Reich 2014), likely because considerable variation exists among species and clades that have evolved to fill different niches within a community in any given habitat (Bruelheide et al. 2018, Treurnicht et al. 2020). As with plants that experience drought stress, plants adapted to colder annual temperatures also tend to produce leaves with higher LMA (Niinemets 2016, Jankowski et al. 2017). However, whether higher LMA is also associated with a greater number of FT cycles or how this trait may vary intraspecifically across the FT and drought poly-stress gradient is unknown. When we compared species’ mean LMA with Di, FTi and PSi, we found that LMA increased with increasing drought (R^2^ = 0.27; *P* < 0.001; Figure 4D), exhibited no relationship with FTi (R^2^ = −0.005; *P* = 0.63; Figure 4E) and increased with increasing poly-stress (R^2^ = 0.12, *P* < 0.001; Figure 4F). This indicates that the response to poly-stress is likely being driven by the strong relationship between LMA and the Di. However, LMA may have stronger relationships with other aspects of FT cycles that our index does not incorporate. For example, our FTi is based on the typical number of FT cycles per year, but it does not account for temperatures prior to or after FT events. Evidence indicates that freezing events can be more damaging (and thus a stronger driver of trait evolution) if they exceed the rate of cold acclimation and/or occur during the deacclimation phase (Sakai 1960, Strimbeck et al. 2007, 2015). It also remains unclear whether relatively minor morphological adjustments result in greater protection to more frequent FT cycles compared with increases in drought stress.

### Traits that impart tolerance in stems

Research on traits that impart tolerance to drought and FT stress in stems has largely focused on maintaining xylem function. Loss of xylem function due to drought stress is caused by the propagation of air bubbles (embolisms) throughout the conduit network. The propagation of an embolism from one tracheid to a neighboring, functional tracheid occurs through the bordered pits on the walls between two tracheids. Theory and empirical evidence support that more drought resistant wood in conifers has a greater overlap between the impermeable portion of the pit membrane, the torus and the pit aperture (Domec et al. 2006, Hacke and Jansen 2009, Delzon et al. 2010, Pittermann et al. 2010, Bouche et al. 2014, Song et al. 2022). As this overlap increases, the water in the functional tracheid must experience increasingly negative pressure to break the pit membrane seal and release a bubble (Cochard et al. 2009). Thus, embolisms propagate throughout the xylem network at less negative pressures in wood containing pits with less overlap.

**Figure 5.**
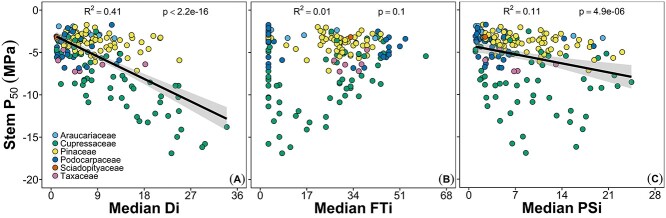
Linear regression of mean species’ stem hydraulic vulnerability (stem P_50_) versus median species’ occurrence on the drought (Di, A), freeze–thaw (FTi, B) and poly-stress (PSi, C) indices. Regression lines and 95% confidence intervals are presented where significant. Each dot represents one species (*n* = 210) in the families indicated.

A commonly used metric to compare the capacity of species to transport water under increasingly negative water potentials is the xylem pressure inducing a 50% loss of hydraulic conductivity in branches (P_50_). Previous work across species has shown that P_50_ values generally become more negative in more arid environments, but that there is considerable variation within a given habitat (Maherali et al. 2004, Choat et al. 2012, McCulloh et al. 2019). When we compared P_50_ values reported in the literature (see Supplemental Methods), we found a relationship consistent with previous results: P_50_ became more negative with increasing Di values (R^2^ = 0.41, *P* < 0.001; Figure 5A). However, the overall relationship is largely driven by data from the Cupressaceae and Taxaceae. The remaining families did not exhibit significant relationships between P_50_ and Di. This distinction highlights the importance of evaluating commonly used metrics such as P_50_ within the context of a species’ physiology. Many Pinaceae species tend to close their stomata at relatively mild water potential values even when living in arid habitats (e.g., *P. edulis*). This conservative approach to maintain favorable water potential values eliminates the need to construct xylem that can continue transporting water under extremely water-stressed conditions.

One of the costs of having stems that can maintain hydraulic function at low water potentials is a necessary increase in wood density. In conifers that function at more negative water potentials, the walls surrounding the tracheids are thicker to prevent collapse during periods of extreme negative pressure and this thickening increases the wood density (Hacke et al. 2001, Hacke and Jansen 2009). Species with high wood density and low capacitance tend to exhibit suites of traits related to drought tolerance more than drought avoidance (McCulloh et al. 2019).

The loss of hydraulic function due to FT-induced embolisms is well documented in conifers (Sperry and Sullivan 1992, Sparks and Black 2000, Feild and Brodribb 2001, Sparks et al. 2001, Mayr et al. 2002, 2003a, 2003*b*, 2007, Pittermann and Sperry 2003, 2006, McCulloh et al. 2011). In contrast to drought stress, interspecific tolerances to FT events are much more directly dependent on tracheid dimensions. During FT cycles, gases that are dissolved in the xylem water will form bubbles due to their insolubility in ice (Hammel 1967, Sucoff 1969). Upon thawing, a bubble has two potential fates: it can dissolve back into the water or it can expand and form an embolism within the xylem (Pittermann and Sperry 2006). The probability of the gas dissolving into the water depends on the size of the bubble, which in turn depends on the diameter of the conduit (and the xylem pressure potential; Pittermann and Sperry 2006). Wider diameter conduits will produce wider diameter bubbles, which will expand at less negative xylem pressures (as described by La Place’s law). Thus, narrower conduits are more resistant to embolism caused by FT cycles, a trend that has been observed across conifers (Pittermann and Sperry 2003, 2006, Willson and Jackson 2006). Freeze–thaw tolerance may also be related to pit characteristics. Theory suggests that pits with greater porosity facilitate the movement of larger particles that allow ice to nucleate at warmer temperatures (Lintunen et al. 2013). Thus, conifers characterized by pits with lower porosity would likely exhibit greater tolerance to FT cycles. Despite the mechanistic link between stem P_50_ and pit dimensions, we found no relationship between P_50_ values and FTi values (R^2^ = 0.01, *P* = 0.1; Figure 5B).

The impact of wood density and capacitance on tolerating FT cycles has not been explored. Theoretically, the high specific heat of water suggests that high water storage in stems may prevent the stem from rapidly freezing or thawing during short periods of low or high temperatures. However, capacitive water that is stored apoplastically would freeze at higher temperatures (compared with symplastically stored water) and could dehydrate the stem symplast, just as the apoplastic water fraction does in leaf tissue (Ball et al. 2002). If so, high density wood with a lower total volume of apoplastically stored water and smaller individual storage compartments may experience less FT damage than low density wood during FT cycles. While wood density has been shown to be negatively correlated with frost tolerance in the woody taxa of North America (Rueda et al. 2017), data are not currently available to determine if these patterns extend to tolerance of FT cycling.

Where the stresses of drought and FT stress overlap, highly poly-tolerant populations would likely have high wood density, narrow conduits and large torus-aperture overlap. Despite some evidence that embolisms induced by FT cycles increase when combined with winter drought, very few studies have measured both FT- and drought-induced embolism on the same species. Loss of hydraulic function in the winter and resistance to drought stress have been examined in four species in the Pacific Northwest of the USA, and no differences were found among the species in either trait (McCulloh et al. 2011, McCulloh et al. 2014). In contrast, found that *Picea abies* lost more hydraulic function than that of neighboring *P. cembra* in the winter but was more resistant to loss of function caused by drought alone. The key difference among these alpine species seems to be the cuticular conductance, which was higher in *P. abies* and caused lower water potential values over the winter than *P. cembra* experienced. If xylem pressure decreases during FT cycles, the probability increases that a gas bubble will expand instead of redissolving, even in narrower conduits (Pittermann and Sperry 2006). Low cuticular and/or minimum stomatal conductance has long been identified as a drought tolerance trait in crops (Sinclair 2000) and has been linked to desiccation tolerance in angiosperms more generally (Gleason et al. 2014, Blackman et al. 2016). A recent review found that 15 *Pinales* species had relatively low values of minimum stomatal conductance compared with angiosperms (Duursma et al. 2019), but it remains unclear the extent to which this trait changes across species ranges in response to greater stress from drought, FT and/or their combination.

Our results also suggest that a more negative P_50_ value imparts some advantage in habitats with higher PSi values (R^2^ = 0.11, *P* < 0.001; Figure 5C). Although this correlation was weak, it was highly significant, and, like the relationship between P_50_ and Di, was driven by trends in the Cupressaceae and Taxaceae. The remaining families did not have significant relationships, although the correlation for the Podocarpaceae suggested a trend (*P* = 0.09). The similarity in the relationships between P_50_ versus Di and PSi suggests that drought tolerance is driving the observed relationship between P_50_ and PSi.

## Discussion and future directions

Our compiled dataset indicates that conifers occupy a large percentage of the worldwide FT and drought poly-stress space (Figure 2), and that individual species differ substantially within this space (Figure 3). Though some species occupy regions with moderate drought and FT stress, no species occupy space with both severe drought and FT stress (Figure 3). Along with the negative relationship of all species’ occurrence along the drought and FT stress indices (R2 = 0.17; *P* < 0.001; data not shown), this distribution suggests a potential functional trade-off in tolerance to these stresses (Figure 3). Although the observed species’ ranges may be limited by other stressors and/or geographic barriers, the lack of data on traits across the FTi, Di and PSi prevents us from fully quantifying the impact of these stresses on distribution patterns. Further analysis of traits across species’ poly-stress ranges may answer whether trait combinations conferring poly-stress tolerance are equivalent for all conifers or if different trait combinations lead to the same tolerance across species.

Our analyses indicate a tendency for individual conifer species to occupy a larger range of FTi than of Di (except when FT tolerance is very low; Figure 3). This tendency remains across all 603 species studied. As a whole, conifers generally occur in habitats with more FT stress than drought stress (Figure 2). These findings may suggest that conifers are better at tolerating FT stress and/or that one unit of the FTi is inherently less stressful than one unit of the Di. This observation drives several questions as follows. In species that occupy a broad range of FT space (e.g., *Callitris rhomboidea* in Figure 3) do traits shift linearly with increasing FT days or does a threshold exist beyond which additional FT days do not correspond to further trait changes? Such a threshold may indicate a diminishing impact of additional FT days. Additionally, while much focus goes toward understanding what allows tolerance to greater stresses, what limits some species occupying locations with lower stress is unknown. Do the traits that provide FT-tolerance have trade-offs that prevent FT tolerant species from competing successfully in low FT areas? Is there a growth penalty for maintaining FT-tolerating traits when FT events are less frequent? Another important point that Figure 3 highlights is the lack of trait data available for many species. Even well-studied species, such as *Pinus sylvestris*, have trait data for locations representing less than half of their distribution along these axes. This lack of data is not unique to these species. Table S1 available as Supplementary data at *Tree Physiology* Online lists all species with their percent trait coverage. Only five species (*Araucaria laubenfelsii*, *Pinus dalatensis*, *Pinus krempfii*, *Pinus koraiensis* and *Retrophyllum minus*) have trait coverage exceeding 50% but these have low occurrences. Many species (76%) have no trait coverage and the median among species with any coverage is 10%. Without a more complete picture of how and if traits vary across species’ ranges, our ability to determine which specific traits are limiting is hindered.

Conifers have a worldwide presence, collectively experiencing a wide range of FT and drought stresses, yet the trait data do not yet exist to fully determine how exactly conifers cope with this poly-stress. For example, the numerous studies that have measured hydraulic vulnerability to drought in a wide range of species contrasts with the comparatively fewer studies that have characterized hydraulic vulnerability to FT cycles. Studies that examine the interaction between low xylem water potential and FT-induced embolism (i.e., the combination of drought and FT cycles) in conifers are even rarer still (Mayr et al. 2006, Pittermann and Sperry 2006, Mayr and Sperry 2010). To help make progress in the study of poly-tolerance in conifers, contrasting high versus low poly-stress ecoregions with abundant conifer species (e.g., Western Turkey sclerophyllous and mixed forests vs Sinaloan dry forests of Mexico) may provide the easiest route to understanding traits conferring FT–drought poly-tolerance.

Finally, it is worth reflecting on the limitation of our analyses regarding future conifer distributions. Rising air temperatures that increase VPD values, unstable atmospheric circulation that drive polar vortices and increased precipitation variability will combine to increase drought, FT and poly-stress globally. The vast boreal forests provide an excellent example of the need for more information on which traits are needed for tolerance to each stress and their combination and what the limits are to those tolerances. Specifically, our FTi, Di and PSi showed that boreal forests currently experience high levels of FT stress but low drought and, consequently, low levels of poly-stress. However, these regions may become drier in the future as altered precipitation patterns result in long, dry periods between summer rain events (Wang et al. 2014). This potential decrease in soil water content will be exacerbated by increases in VPD, which would intensify the drought stress (Yuan et al. 2019, López et al. 2021). These changes will increase the occurrence and intensity of poly-stress, and thus, a tolerance to both FT and drought will be critical for the survival and growth of coniferous species at these latitudes. Consequently, we need a more fundamental understanding of the traits that permit tolerance of these stresses for a broader range of species across their distributions to predict how these forests will respond to FT and drought stress in the future.

## Conclusion

In this review, we present four key points: (i) the global peak co-occurrence of drought and FT stresses is the combination of moderate drought and moderate FT; (ii) the distribution of conifers overlaps considerably with moderate levels of FT and drought stress; (iii) the distribution of individual species along the two indices varies, with some species exhibiting broad tolerance to both stresses and others exhibiting tolerance to only one (typically FT); and (iv) while several hydraulic traits may confer tolerance to both FT cycles and drought in stems and leaves, the lack of trait data across species ranges limits our understanding of poly-tolerance in conifers globally. Given that global climate change will likely increase the frequency and severity of both FT cycles and drought, addressing this knowledge gap will be of critical importance for predicting how coniferous forests will function in the future.

The emergent science of poly-tolerance is primarily limited by the scarcity of comprehensive intra- and interspecific datasets across broad geographical scales. Many hydraulic traits that may be involved in poly-tolerance—such as conduit and pit dimensions or TLP—are typically measured in a few species at small spatial scales due to the time-intensive nature of these measurements. Recent efforts to expedite these measurements (e.g., measuring TLP by osmometry; Bartlett et al. 2012) have been successful and will facilitate the broad-scale survey of these traits across ecological gradients, especially with increasing utilization of global field experiment networks such as DroughtNet (Knapp et al. 2015) and the Center for Forest Global Earth Observatory ‘ForestGeo’ (Davies et al. 2021). Furthermore, the observed distribution of species across these indices suggests that different species may have varying levels of tolerance to each stress, indicating the response of conifers to increasing poly-stress will likely be species-specific. Coniferous forests dominated by species that have low poly-tolerance to these stressors may consequently experience range shifts or extirpation in the future. Drastic changes in the distribution of conifer species will have far-reaching industrial, agricultural and cultural implications, underscoring the need to strengthen poly-tolerance concepts.

## Supplementary Material

S1_ft-day_vs_tmin_v2_tpac102

SFig2_tpac102

S3_tpac102

fig_S4_tpac102

Supplemental_Table1_tpac102

Supplementary_Table_S2_tpac102

Supplemental_materials_final_tpac102

## References

[ref1] Abatzoglou JT, Dobrowski SZ, Parks SA, Hegewisch KC (2018) TerraClimate, a high-resolution global dataset of monthly climate and climatic water balance from 1958–2015. Sci Data 5:1–12. 29313841 10.1038/sdata.2017.191 PMC5759372

[ref2] Allen CD, Macalady AK, Chenchouni H, Bachelet D, McDowell N, Vennetier M, Gonzalez P (2010) A global overview of drought and heat-induced tree mortality reveals emerging climate change risks for forests. For Ecol Manage 259:660–684.

[ref3] Allen DJ, Ort DR (2001) Impacts of chilling temperatures on photosynthesis in warm-climate plants. Trends Plant Sci 6:36–42. 11164376 10.1016/s1360-1385(00)01808-2

[ref4] Ambroise V, Legay S, Guerriero G, Hausman JF, Cuypers A, Sergeant K (2020) The roots of plant frost hardiness and tolerance. Plant Cell Physiol 61:3–20. 31626277 10.1093/pcp/pcz196 PMC6977023

[ref5] Anderegg WR, Kane JM, Anderegg LD (2013) Consequences of widespread tree mortality triggered by drought and temperature stress. Nat Clim Change 3:30–36.

[ref6] Anderegg WR, Klein T, Bartlett M, Sack L, Pellegrini AF, Choat B, Jansen S (2016) Meta-analysis reveals that hydraulic traits explain cross-species patterns of drought-induced tree mortality across the globe. Proc Natl Acad Sci USA 113:5024–5029. 27091965 10.1073/pnas.1525678113 PMC4983847

[ref7] Arias NS, Bucci SJ, Scholz FG, Goldstein G (2015) Freezing avoidance by supercooling in *Olea europaea* cultivars: the role of apoplastic water, solute content and cell wall rigidity. Plant Cell Environ 38:2061–2070. 25737264 10.1111/pce.12529

[ref8] Arias NS, Scholz FG, Goldstein G, Bucci SJ (2017) The cost of avoiding freezing in stems: trade-off between xylem resistance to cavitation and supercooling capacity in woody plants. Tree Physiol 37:1251–1262. 28633378 10.1093/treephys/tpx071

[ref9] Ball MC, Wolfe J, Canny M, Hofmann M, Nicotra AB, Hughes D (2002) Space and time dependence of temperature and freezing in evergreen leaves. Funct Plant Biol 29:1259–1272. 32688724 10.1071/FP02037

[ref10] Bartlett MK, Scoffoni C, Sack L (2012) The determinants of leaf turgor loss point and prediction of drought tolerance of species and biomes: a global meta-analysis. Ecol Lett 15:393–405. 22435987 10.1111/j.1461-0248.2012.01751.x

[ref12] Blackman CJ, Pfautsch S, Choat B, Delzon S, Gleason SM, Duursma RA (2016) Toward an index of desiccation time to tree mortality under drought. Plant Cell Environ 39:2342–2345. 27093688 10.1111/pce.12758

[ref11] Blackman CJ, Creek D, Maier C, Aspinwall MJ, Drake JE, Pfautsch S, Choat B (2019) Drought response strategies and hydraulic traits contribute to mechanistic understanding of plant dry-down to hydraulic failure. Tree Physiol 39:910–924. 30865274 10.1093/treephys/tpz016

[ref13] Bouche PS, Larter M, Domec JC, Burlett R, Gasson P, Jansen S, Delzon S (2014) A broad survey of hydraulic and mechanical safety in the xylem of conifers. J Exp Bot 65:4419–4431. 24916072 10.1093/jxb/eru218 PMC4112641

[ref14] Brodribb TJ, Cochard H (2009) Hydraulic failure defines the recovery and point of death in water-stressed conifers. Plant Physiol 149:575–584. 19011001 10.1104/pp.108.129783 PMC2613726

[ref15] Brodribb TJ, Holbrook NM (2003) Stomatal closure during leaf dehydration, correlation with other leaf physiological traits. Plant Physiol 132:2166–2173. 12913171 10.1104/pp.103.023879 PMC181300

[ref16] Brodribb TJ, Holbrook NM (2006) Declining hydraulic efficiency as transpiring leaves desiccate: two types of response. Plant Cell Environ 29:2205–2215. 17081253 10.1111/j.1365-3040.2006.01594.x

[ref17] Bruelheide H, Dengler J, Purschke O, Lenoir J, Jiménez-Alfaro B, Hennekens SM, Jandt U (2018) Global trait–environment relationships of plant communities. Nat Ecol Evol 2:1906–1917. 30455437 10.1038/s41559-018-0699-8

[ref18] Casson NJ, Contosta AR, Burakowski EA, Campbell JL, Crandall MS, Creed IF, Nelson SJ (2019) Winter weather whiplash: impacts of meteorological events misaligned with natural and human systems in seasonally snow-covered regions. Earths Future 7:1434–1450.

[ref21] Chang CYY, Bräutigam K, Hüner NP, Ensminger I (2021) Champions of winter survival: cold acclimation and molecular regulation of cold hardiness in evergreen conifers. New Phytol 229:675–691. 32869329 10.1111/nph.16904

[ref22] Charrier G, Cochard H, Améglio T (2013) Evaluation of the impact of frost resistances on potential altitudinal limit of trees. Tree Physiol 33:891–902. 24052567 10.1093/treephys/tpt062

[ref23] Charrier G, Martin-StPaul N, Damesin C, Delpierre N, Hänninen H, Torres-Ruiz JM, Davi H (2021) Interaction of drought and frost in tree ecophysiology: rethinking the timing of risks. Ann For Sci 78:40.

[ref25] Choat B, Jansen S, Brodribb TJ, Cochard H, Delzon S, Bhaskar R, Zanne AE (2012) Global convergence in the vulnerability of forests to drought. Nature 491:752–755. 23172141 10.1038/nature11688

[ref24] Choat B, Brodribb TJ, Brodersen CR, Duursma RA, Lopez R, Medlyn BE (2018) Triggers of tree mortality under drought. Nature 558:531–539. 29950621 10.1038/s41586-018-0240-x

[ref26] Clark JS, Iverson L, Woodall CW, Allen CD, Bell DM, Bragg DC, Zimmermann NE (2016) The impacts of increasing drought on forest dynamics, structure, and biodiversity in the United States. Glob Chang Biol 22:2329–2352. 26898361 10.1111/gcb.13160

[ref27] Clow DW (2010) Changes in the timing of snowmelt and streamflow in Colorado: a response to recent warming. J Clim 23:2293–2306.

[ref28] Cochard H, Hölttä T, Herbette S, Delzon S, Mencuccini M (2009) New insights into the mechanisms of water-stress-induced cavitation in conifers. Plant Physiol 151:949–954. 19641033 10.1104/pp.109.138305 PMC2754640

[ref29] Dai A, Zhao T, Chen J (2018) Climate change and drought: a precipitation and evaporation perspective. Curr Clim Change Rep 4:301–312.

[ref30] Davies SJ, Abiem I, Salim KA, Aguilar S, Allen D, Alonso A, Yap SL (2021) ForestGEO: understanding forest diversity and dynamics through a global observatory network. Biol Conserv 253:108907.

[ref31] Delzon S, Douthe C, Sala A, Cochard H (2010) Mechanism of water-stress induced cavitation in conifers: bordered pit structure and function support the hypothesis of seal capillary-seeding. Plant Cell Environ 33:2101–2111. 20636490 10.1111/j.1365-3040.2010.02208.x PMC3003904

[ref32] Demmig-Adams B, Adams WW III (2006) Photoprotection in an ecological context: the remarkable complexity of thermal energy dissipation. New Phytol 172:11–21. 16945085 10.1111/j.1469-8137.2006.01835.x

[ref34] Domec JC, Lachenbruch B, Meinzer FC (2006) Bordered pit structure and function determine spatial patterns of air-seeding thresholds in xylem of Douglas-fir (*Pseudotsuga menziesii*; Pinaceae) trees. Am J Bot 93:1588–1600. 21642104 10.3732/ajb.93.11.1588

[ref35] Duursma RA, Blackman CJ, Lopéz R, Martin-StPaul NK, Cochard H, Medlyn BE (2019) On the minimum leaf conductance: its role in models of plant water use, and ecological and environmental controls. New Phytol 221:693–705. 30144393 10.1111/nph.15395

[ref36] Earles JM, Stevens JT, Sperling O, Orozco J, North MP, Zwieniecki MA (2018) Extreme mid-winter drought weakens tree hydraulic–carbohydrate systems and slows growth. New Phytol 219:89–97. 29663406 10.1111/nph.15136

[ref37] Engelbrecht BM, Comita LS, Condit R, Kursar TA, Tyree MT, Turner BL, Hubbell SP (2007) Drought sensitivity shapes species distribution patterns in tropical forests. Nature 447:80–82. 17476266 10.1038/nature05747

[ref39] Feild TS, Brodribb T (2001) Stem water transport and freeze-thaw xylem embolism in conifers and angiosperms in a Tasmanian treeline heath. Oecologia 127:314–320. 28547101 10.1007/s004420000603

[ref40] Givnish TJ (2002) Adaptive significance of evergreen vs. deciduous leaves: solving the triple paradox. Silva Fenn 36:703–743.

[ref41] Gleason SM, Blackman CJ, Cook AM, Laws CA, Westoby M (2014) Whole-plant capacitance, embolism resistance and slow transpiration rates all contribute to longer desiccation times in woody angiosperms from arid and wet habitats. Tree Physiol 34:275–284. 24550089 10.1093/treephys/tpu001

[ref42] Goldstein G, Rada F, Azocar A (1985) Cold hardiness and supercooling along an altitudinal gradient in Andean giant rosette species. Oecologia 68:147–152. 28310924 10.1007/BF00379487

[ref44] Gross K, Koch W (1991) Water relations of *Picea abies*. I. Comparison of water relations parameters of spruce shoots examined at the end of the vegetation period and in winter. Physiol Plant 83:290–295.

[ref45] Grossnickle SC (1992) Relationship between freezing tolerance and shoot water relations of western red cedar. Tree Physiol 11:229–240. 14969948 10.1093/treephys/11.3.229

[ref46] Guy CL (1990) Cold acclimation and freezing stress tolerance: role of protein metabolism. Annu Rev Plant Biol 41:187–223.

[ref47] Guy CL (2003) Freezing tolerance of plants: current understanding and selected emerging concepts. Can J Bot 81:1216–1223.

[ref48] Hacke UG, Jansen S (2009) Embolism resistance of three boreal conifer species varies with pit structure. New Phytol 182:675–686. 19309447 10.1111/j.1469-8137.2009.02783.x

[ref49] Hacke UG, Sperry JS, Pockman WT, Davis SD, McCulloh KA (2001) Trends in wood density and structure are linked to prevention of xylem implosion by negative pressure. Oecologia 126:457–461. 28547229 10.1007/s004420100628

[ref50] Hallik L, Niinemets Ü, Wright IJ (2009) Are species shade and drought tolerance reflected in leaf-level structural and functional differentiation in Northern Hemisphere temperate woody flora? New Phytol 184:257–274. 19674334 10.1111/j.1469-8137.2009.02918.x

[ref51] Hammel HT (1967) Freezing of xylem sap without cavitation. Plant Physiol 42:55–66. 16656485 10.1104/pp.42.1.55 PMC1086489

[ref52] Henry HA (2008) Climate change and soil freezing dynamics: historical trends and projected changes. Clim Change 87:421–434.

[ref53] Hodgson JG, Montserrat-Martí G, Charles M, Jones G, Wilson P, Shipley B, Royo Pla F (2011) Is leaf dry matter content a better predictor of soil fertility than specific leaf area? Ann Bot 108:1337–1345. 21948627 10.1093/aob/mcr225 PMC3197453

[ref54] Hu JIA, Moore DJ, Burns SP, Monson RK (2010) Longer growing seasons lead to less carbon sequestration by a subalpine forest. Glob Chang Biol 16:771–783.

[ref56] Huner NP, Öquist G, Sarhan F (1998) Energy balance and acclimation to light and cold. Trends Plant Sci 3:224–230.

[ref55] Huner, NP, Öquist, G, & Melis, A (2003). Photostasis in plants, green algae and cyanobacteria: the role of light harvesting antenna complexes. In: Green, B.R., Parson, W.W. (eds). Light-harvesting antennas in photosynthesis. Advances in Photosynthesis and Respiration, vol 13. Springer, Dordrecht, pp. 401–421. 10.1007/978-94-017-2087-8_14.

[ref57] IPCC (2019) Climate Change and Land: an IPCC special report on climate change, desertification, land degradation, sustainable land management, food security, and greenhouse gas fluxes in terrestrial ecosystems. In: Shukla PR, Skea J, Buendia EC et al. (eds). https://www.ipcc.ch/srccl/cite-report/#:~:text=This%20report%20should%20be%20cited,Skea%2C%20E.

[ref58] Ivanov A, Sane P, Zeinalov Y, Malmberg G, Gardeström P, Huner N, Öquist G (2001) Photosynthetic electron transport adjustments in overwintering Scots pine (*Pinus sylvestris* L.). Planta 213:575–585. 11556790 10.1007/s004250100522

[ref59] Jankowski A, Wyka TP, Żytkowiak R, Nihlgård B, Reich PB, Oleksyn J (2017) Cold adaptation drives variability in needle structure and anatomy in *Pinus sylvestris* L. along a 1,900 km temperate–boreal transect. Funct Ecol 31:2212–2223.

[ref60] Johnson DM, Berry ZC, Baker KV, Smith DD, McCulloh KA, Domec JC (2018) Leaf hydraulic parameters are more plastic in species that experience a wider range of leaf water potentials. Funct Ecol 32:894–903.

[ref62] Kasuga J, Arakawa K, Fujikawa S (2007) High accumulation of soluble sugars in deep supercooling Japanese white birch xylem parenchyma cells. New Phytol 174:569–579. 17447912 10.1111/j.1469-8137.2007.02025.x

[ref64] Knapp AK, Hoover DL, Wilcox KR, Avolio ML, Koerner SE, La Pierre KJ, Loik ME, Lou Y, Sala OE, Smith MD (2015) Characterizing differences in precipitation regimes of extreme wet and dry years: implications for climate change experiments. Glob Chang Biol 21:2624–2633. 25652911 10.1111/gcb.12888

[ref65] Knowles N, Dettinger MD, Cayan DR (2006) Trends in snowfall versus rainfall in the western United States. J Clim 19:4545–4559.

[ref66] Kreyling J (2010) Winter climate change: a critical factor for temperate vegetation performance. Ecology 91:1939–1948. 20715613 10.1890/09-1160.1

[ref67] Laanisto L, Niinemets Ü (2015) Polytolerance to abiotic stresses: how universal is the shade–drought tolerance trade-off in woody species? Glob Ecol Biogeogr 24:571–580. 29367836 10.1111/geb.12288 PMC5777592

[ref69] Larter M, Brodribb TJ, Pfautsch S, Burlett R, Cochard H, Delzon S (2015) Extreme aridity pushes trees to their physical limits. Plant Physiol 168:804–807. 26034263 10.1104/pp.15.00223 PMC4741339

[ref70] Larter M, Pfautsch S, Domec JC, Trueba S, Nagalingum N, Delzon S (2017) Aridity drove the evolution of extreme embolism resistance and the radiation of conifer genus *Callitris*. New Phytol 215:97–112. 28378882 10.1111/nph.14545

[ref71] Le Gall H, Philippe F, Domon JM, Gillet F, Pelloux J, Rayon C (2015) Cell wall metabolism in response to abiotic stress. Plan Theory 4:112–166. 10.3390/plants4010112 PMC4844334 27135320

[ref72] Li X, Blackman CJ, Choat B, Duursma RA, Rymer PD, Medlyn BE, Tissue DT (2018) Tree hydraulic traits are coordinated and strongly linked to climate-of-origin across a rainfall gradient. Plant Cell Environ 41:646–660. 29314083 10.1111/pce.13129

[ref73] Lintunen A, Hölttä T, Kulmala M (2013) Anatomical regulation of ice nucleation and cavitation helps trees to survive freezing and drought stress. Sci Rep 3:1–7. 10.1038/srep02031 PMC3686780 23778457

[ref74] Lintunen A, Mayr S, Salmon Y, Cochard H, Hölttä T (2018) Drivers of apoplastic freezing in gymnosperm and angiosperm branches. Ecol Evol 8:333–343. 29321875 10.1002/ece3.3665 PMC5756836

[ref75] López J, Way DA, Sadok W (2021) Systemic effects of rising atmospheric vapor pressure deficit on plant physiology and productivity. Glob Chang Biol 27:1704–1720. 33683792 10.1111/gcb.15548 PMC8251766

[ref76] Maherali H, Pockman WT, Jackson RB (2004) Adaptive variation in the vulnerability of woody plants to xylem cavitation. Ecology 85:2184–2199.

[ref77] Matsumura S, Yamazaki K, Horinouchi T (2021) Robust asymmetry of the future Arctic polar vortex is driven by tropical Pacific warming. Geophys Res Lett 48:e2021GL093440. 10.1029/2021GL093440.

[ref78] Mayr S, Améglio T (2016) Freezing stress in tree xylem. In: Lüttge, U., Cánovas, F.M., Matyssek, R. (eds). 77, 77, Springer International Publishing, 435 p., 10.1007/978-3-319-25688-7_13.

[ref83] Mayr S, Sperry JS (2010) Freeze–thaw-induced embolism in *Pinus contorta*: centrifuge experiments validate the ‘thaw-expansion hypothesis’ but conflict with ultrasonic emission data. New Phytol 185:1016–1024. 20028475 10.1111/j.1469-8137.2009.03133.x

[ref84] Mayr S, Wolfschwenger M, Bauer H (2002) Winter-drought induced embolism in Norway spruce (*Picea abies*) at the Alpine timberline. Physiol Plant 115:74–80. 12010469 10.1034/j.1399-3054.2002.1150108.x

[ref82] Mayr S, Schwienbacher F, Bauer H (2003 *a*) Winter at the alpine timberline. Why does embolism occur in Norway spruce but not in stone pine? Plant Physiol 131:780–792. 12586902 10.1104/pp.011452 PMC166854

[ref80] Mayr S, Gruber A, Bauer H (2003 *b*) Repeated freeze–thaw cycles induce embolism in drought stressed conifers (Norway spruce, stone pine). Planta 217:436–441. 14520570 10.1007/s00425-003-0997-4

[ref81] Mayr S, Hacke U, Schmid P, Schwienbacher F, Gruber A (2006) Frost drought in conifers at the alpine timberline: xylem dysfunction and adaptations. Ecology 87:3175–3185. 17249241 10.1890/0012-9658(2006)87[3175:fdicat]2.0.co;2

[ref79] Mayr S, Cochard H, Améglio T, Kikuta SB (2007) Embolism formation during freezing in the wood of *Picea abies*. Plant Physiol 143:60–67. 17041033 10.1104/pp.106.085704 PMC1761990

[ref87] McCulloh KA, Johnson DM, Meinzer FC, Lachenbruch B (2011) An annual pattern of native embolism in upper branches of four tall conifer species. Am J Bot 98:1007–1015. 21613067 10.3732/ajb.1000503

[ref88] McCulloh KA, Johnson DM, Meinzer FC, Woodruff DR (2014) The dynamic pipeline: hydraulic capacitance and xylem hydraulic safety in four tall conifer species. Plant Cell Environ 37:1171–1183. 24289816 10.1111/pce.12225

[ref86] McCulloh KA, Domec JC, Johnson DM, Smith DD, Meinzer FC (2019) A dynamic yet vulnerable pipeline: integration and coordination of hydraulic traits across whole plants. Plant Cell Environ 42:2789–2807. 31273812 10.1111/pce.13607

[ref89] McDowell N, Pockman WT, Allen CD, Breshears DD, Cobb N, Kolb T, Yepez EA (2008) Mechanisms of plant survival and mortality during drought: why do some plants survive while others succumb to drought? New Phytol 178:719–739. 18422905 10.1111/j.1469-8137.2008.02436.x

[ref90] Meinzer FC, Campanello PI, Domec JC, Gatti MG, Goldstein G, Villalobos-Vega R, Woodruff DR (2008) Constraints on physiological function associated with branch architecture and wood density in tropical forest trees. Tree Physiol 28:1609–1617. 18765366 10.1093/treephys/28.11.1609

[ref91] Meinzer FC, Woodruff DR, Marias DE, McCulloh KA, Sevanto S (2014) Dynamics of leaf water relations components in co-occurring iso-and anisohydric conifer species. Plant Cell Environ 37:2577–2586. 24661116 10.1111/pce.12327

[ref92] Mote PW, Hamlet AF, Clark MP, Lettenmaier DP (2005) Declining mountain snowpack in western North America. Bull Am Meteorol Soc 86:39–50.

[ref93] Nardini A, Luglio J (2014) Leaf hydraulic capacity and drought vulnerability: possible trade- offs and correlations with climate across three major biomes. Funct Ecol 28:810–818.

[ref94] Nardini A, Pedà G, Rocca NL (2012) Trade-offs between leaf hydraulic capacity and drought vulnerability: morpho-anatomical bases, carbon costs and ecological consequences. New Phytol 196:788–798. 22978628 10.1111/j.1469-8137.2012.04294.x

[ref95] Niinemets Ü (2016) Does the touch of cold make evergreen leaves tougher? Tree Physiol 36:267–272. 26917702 10.1093/treephys/tpw007 PMC4885950

[ref96] Normand S, Treier UA, Randin C, Vittoz P, Guisan A, Svenning JC (2009) Importance of abiotic stress as a range-limit determinant for European plants: insights from species responses to climatic gradients. Glob Ecol Biogeogr 18:437–449.

[ref99] Pittermann J, Sperry J (2003) Tracheid diameter is the key trait determining the extent of freezing-induced embolism in conifers. Tree Physiol 23:907–914. 14532014 10.1093/treephys/23.13.907

[ref100] Pittermann J, Sperry JS (2006) Analysis of freeze-thaw embolism in conifers. The interaction between cavitation pressure and tracheid size. Plant Physiol 140:374–382. 16377751 10.1104/pp.105.067900 PMC1326058

[ref98] Pittermann J, Choat B, Jansen S, Stuart SA, Lynn L, Dawson TE (2010) The relationships between xylem safety and hydraulic efficiency in the Cupressaceae: the evolution of pit membrane form and function. Plant Physiol 153:1919–1931. 20551212 10.1104/pp.110.158824 PMC2923884

[ref102] Reich PB (2014) The world-wide ‘fast–slow’plant economics spectrum: a traits manifesto. J Ecol 102:275–301.

[ref103] Ritchie GA, Shula RG (1984) Seasonal changes of tissue-water relations in shoots and root systems of Douglas-fir seedlings. Forest Sci 30:538–548.

[ref104] Rosas T, Mencuccini M, Barba J, Cochard H, Saura-Mas S, Martínez-Vilalta J (2019) Adjustments and coordination of hydraulic, leaf and stem traits along a water availability gradient. New Phytol 223:632–646. 30636323 10.1111/nph.15684

[ref105] Rosas T, Mencuccini M, Batlles C, Regalado Í, Saura-Mas S, Sterck F, Martínez-Vilalta J (2021) Are leaf, stem and hydraulic traits good predictors of individual tree growth? Funct Ecol 35:2435–2447.

[ref107] Rueda M, Godoy O, Hawkins BA (2017) Spatial and evolutionary parallelism between shade and drought tolerance explains the distributions of conifers in the conterminous United States. Glob Ecol Biogeogr 26:31–42.

[ref108] Saito T, Terashima I (2004) Reversible decreases in the bulk elastic modulus of mature leaves of deciduous *Quercus* species subjected to two drought treatments. Plant Cell Environ 27:863–875.

[ref109] Sakai A (1960) Survival of the twig of woody plants at −196°C. Nature 185:393–394.

[ref110] Sakai A, Larcher W (2012) Frost survival of plants: responses and adaptation to freezing stress, Vol. 62. Springer Berlin, Heidelberg: Springer Science & Business Media.

[ref111] Sanchez-Martinez P, Martínez-Vilalta J, Dexter KG, Segovia RA, Mencuccini M (2020) Adaptation and coordinated evolution of plant hydraulic traits. Ecol Lett 23:1599–1610. 32808458 10.1111/ele.13584

[ref112] Scholz FG, Bucci SJ, Arias N, Meinzer FC, Goldstein G (2012) Osmotic and elastic adjustments in cold desert shrubs differing in rooting depth: coping with drought and subzero temperatures. Oecologia 170:885–897. 22644052 10.1007/s00442-012-2368-y

[ref113] Sebastian-Azcona J, Hacke UG, Hamann A (2018) Adaptations of white spruce to climate: strong intraspecific differences in cold hardiness linked to survival. Ecol Evol 8:1758–1768. 29435250 10.1002/ece3.3796 PMC5792524

[ref114] Simonin KA, Limm EB, Dawson TE (2012) Hydraulic conductance of leaves correlates with leaf lifespan: implications for lifetime carbon gain. New Phytol 193:939–947. 22224403 10.1111/j.1469-8137.2011.04014.x

[ref115] Sinclair TR (2000) Model analysis of plant traits leading to prolonged crop survival during severe drought. Field Crop Res 68:211–217.

[ref116] Song Y, Poorter L, Horsting A, Delzon S, Sterck F (2022) Pit and tracheid anatomy explain hydraulic safety but not hydraulic efficiency of 28 conifer species. J Exp Bot 73:1033–1048. 34626106 10.1093/jxb/erab449 PMC8793876

[ref117] Sparks JP, Black RA (2000) Winter hydraulic conductivity and xylem cavitation in coniferous trees from upper and lower treeline. Arctic Antarct Alpine Res 32:397–403.

[ref118] Sparks JP, Campbell GS, Black AR (2001) Water content, hydraulic conductivity, and ice formation in winter stems of *Pinus contorta*: a TDR case study. Oecologia 127:468–475. 28547483 10.1007/s004420000587

[ref119] Sperry JS, Sullivan JE (1992) Xylem embolism in response to freeze-thaw cycles and water stress in ring-porous, diffuse-porous, and conifer species. Plant Physiol 100:605–613. 16653035 10.1104/pp.100.2.605 PMC1075601

[ref121] Stahl U, Kattge J, Reu B, Voigt W, Ogle K, Dickie J, Wirth C (2013) Whole-plant trait spectra of North American woody plant species reflect fundamental ecological strategies. Ecosphere 4:art128. 10.1890/ES13-00143.1.

[ref122] Stahl U, Reu B, Wirth C (2014) Predicting species’ range limits from functional traits for the tree flora of North America. Proc Natl Acad Sci USA 111:13739–13744. 25225398 10.1073/pnas.1300673111 PMC4183311

[ref123] Strimbeck GR, Kjellsen TD, Schaberg PG, Murakami PF (2007) Cold in the common garden: comparative low-temperature tolerance of boreal and temperate conifer foliage. Trees 21:557–567.

[ref124] Strimbeck GR, Schaberg PG, Fossdal CG, Schröder WP, Kjellsen TD (2015) Extreme low temperature tolerance in woody plants. Front Plant Sci 6:884. 10.3389/fpls.2015.00884. 26539202 PMC4609829

[ref125] Stuart SA, Choat B, Martin KC, Holbrook NM, Ball MC (2007) The role of freezing in setting the latitudinal limits of mangrove forests. New Phytol 173:576–583. 17244052 10.1111/j.1469-8137.2006.01938.x

[ref126] Sucoff E (1969) Freezing of conifer xylem and the cohesion-tension theory. Physiol Plant 22:424–431.

[ref127] Sundaram M, Donoghue MJ, Farjon A, Filer D, Mathews S, Jetz W, Leslie AB (2019) Accumulation over evolutionary time as a major cause of biodiversity hotspots in conifers. Proc R Soc B 286:20191887. 10.1098/rspb.2019.1887. PMC6790781 31594500

[ref128] Ting CH, Mycock DJ, Padayachee K (2014) Cold pretreatment amplifies the responses of in vitro *Eucalyptus grandis* shoots to cryopreparative drying. Cryo Letters 35:54–62. 24872158

[ref129] Tranquillini W (1976) Water relations and alpine timberline. In Water and Plant Life. Springer, Berlin, Heidelberg, pp. 473–491. 10.1007/978-3-642-66429-8_29.

[ref130] Treurnicht M, Pagel J, Tonnabel J, Esler KJ, Slingsby JA, Schurr FM (2020) Functional traits explain the Hutchinsonian niches of plant species. Glob Ecol Biogeogr 29:534–545.

[ref132] Tyree MT, Sperry JS (1989) Vulnerability of xylem to cavitation and embolism. Annu Rev Plant Biol 40:19–36.

[ref133] Tyree MT, Zimmermann MH (2013) Xylem structure and the ascent of sap. Springer Science & Business Media.

[ref131] Tyree MT, Cheung YNS, MacGregor ME, Talbot AJB (1978) The characteristics of seasonal and ontogenetic changes in the tissue–water relations of *Acer*, *Populus*, *Tsuga*, and *Picea*. Can J Bot 56:635–647.

[ref134] Wang Y, Hogg EH, Price DT, Edwards J, Williamson T (2014) Past and projected future changes in moisture conditions in the Canadian boreal forest. Forestry Chronicle 90:678–691.

[ref135] Williams CM, Henry HA, Sinclair BJ (2015) Cold truths: how winter drives responses of terrestrial organisms to climate change. Biol Rev 90:214–235. 24720862 10.1111/brv.12105

[ref136] Willson CJ, Jackson RB (2006) Xylem cavitation caused by drought and freezing stress in four co-occurring *Juniperus* species. Physiol Plant 127:374–382.

[ref137] Wisniewski M, Nassuth A, Arora R (2018) Cold hardiness in trees: a mini-review. Front Plant Sci 9:1394. 10.3389/fpls.2018.01394. 30294340 PMC6158558

[ref138] Xin Z, Browse J (2000) Cold comfort farm: the acclimation of plants to freezing temperatures. Plant Cell Environ 23:893–902.

[ref139] Yao GQ, Nie ZF, Turner NC, Li FM, Gao TP, Fang XW, Scoffoni C (2021) Combined high leaf hydraulic safety and efficiency provides drought tolerance in *Caragana* species adapted to low mean annual precipitation. New Phytol 229:230–244. 32749703 10.1111/nph.16845 PMC7754512

[ref140] Yuan W, Zheng Y, Piao S, Ciais P, Lombardozzi D, Wang Y, Yang S (2019) Increased atmospheric vapor pressure deficit reduces global vegetation growth. Sci Adv 5:eaax1396. 10.1126/sciadv.aax1396. PMC6693914 31453338

[ref142] Zanne AE, Tank DC, Cornwell WK, Eastman JM, Smith SA, FitzJohn RG, Beaulieu JM (2014) Three keys to the radiation of angiosperms into freezing environments. Nature 506:89–92. 24362564 10.1038/nature12872

[ref141] Zanne AE, Pearse WD, Cornwell WK, McGlinn DJ, Wright IJ, Uyeda JC (2018) Functional biogeography of angiosperms: life at the extremes. New Phytol 218:1697–1709. 29603243 10.1111/nph.15114

[ref143] Zhang J, Tian W, Chipperfield MP, Xie F, Huang J (2016) Persistent shift of the Arctic polar vortex towards the Eurasian continent in recent decades. Nat Clim Change 6:1094–1099.

[ref144] Zhang YJ, Bucci SJ, Arias NS, Scholz FG, Hao GY, Cao KF, Goldstein G (2016) Freezing resistance in Patagonian woody shrubs: the role of cell wall elasticity and stem vessel size. Tree Physiol 36:1007–1018. 27217529 10.1093/treephys/tpw036

