# Supplementary figures and images for: At least it is a dry cold: the global distribution of freeze–thaw and drought stress and the traits that may impart poly-tolerance in conifers
Source: Tree Physiol. 2022 Sep 12;43(1):1–15. doi: 10.1093/treephys/tpac102 (PMC9833871; doi:10.1093/treephys/tpac102)

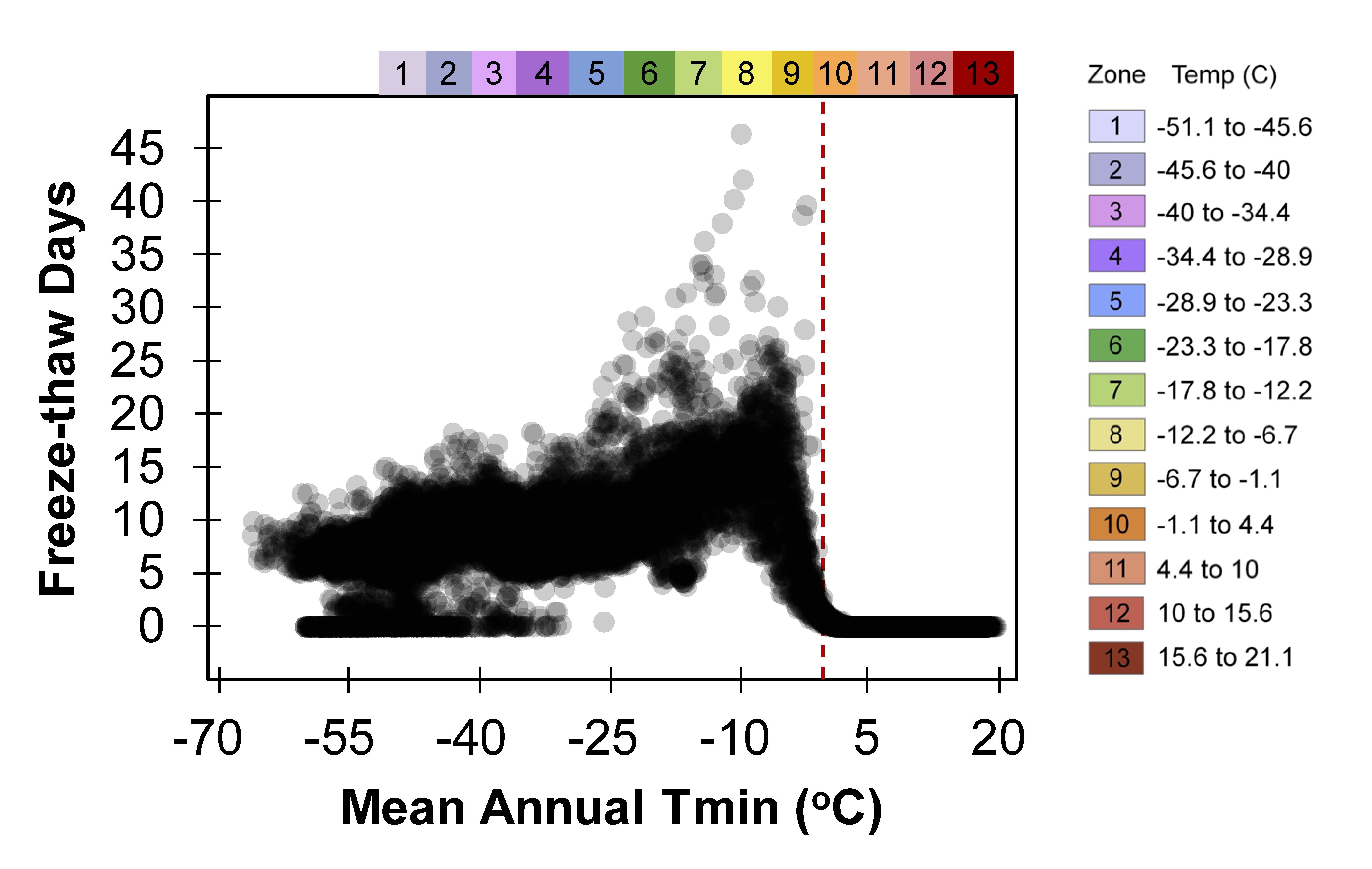

Supplement: S1_ft-day_vs_tmin_v2_tpac102 [file s1_ft-day_vs_tmin_v2_tpac102.jpeg]

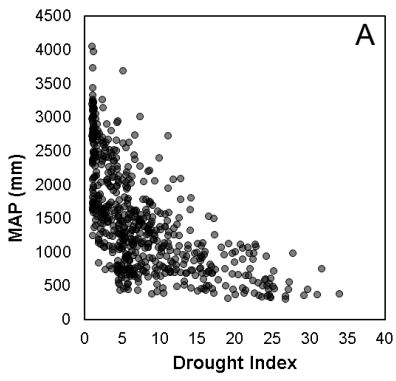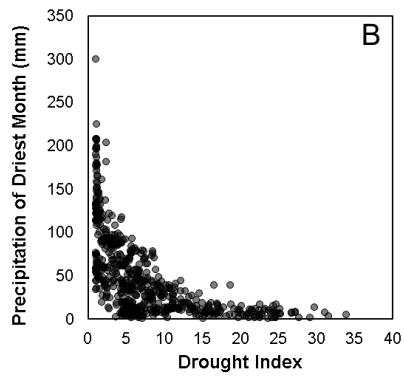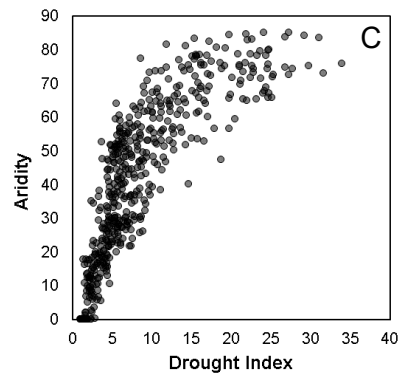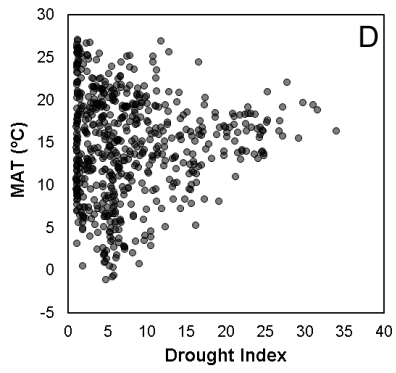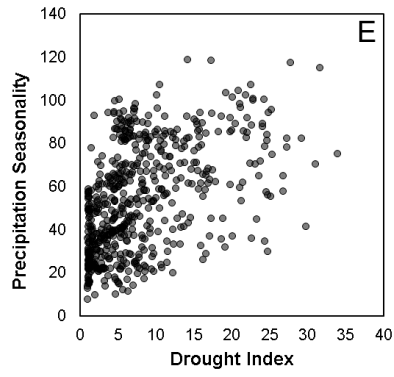

Supplement: SFig2_tpac102 [file sfig2_tpac102.pdf]

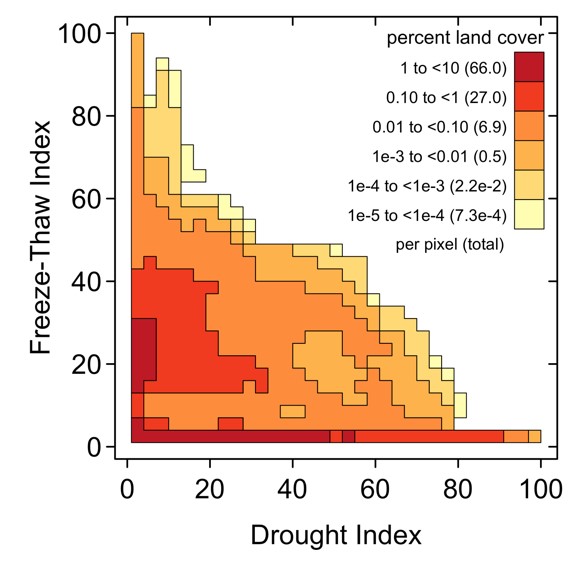

Supplement: S3_tpac102 [file s3_tpac102.jpeg]

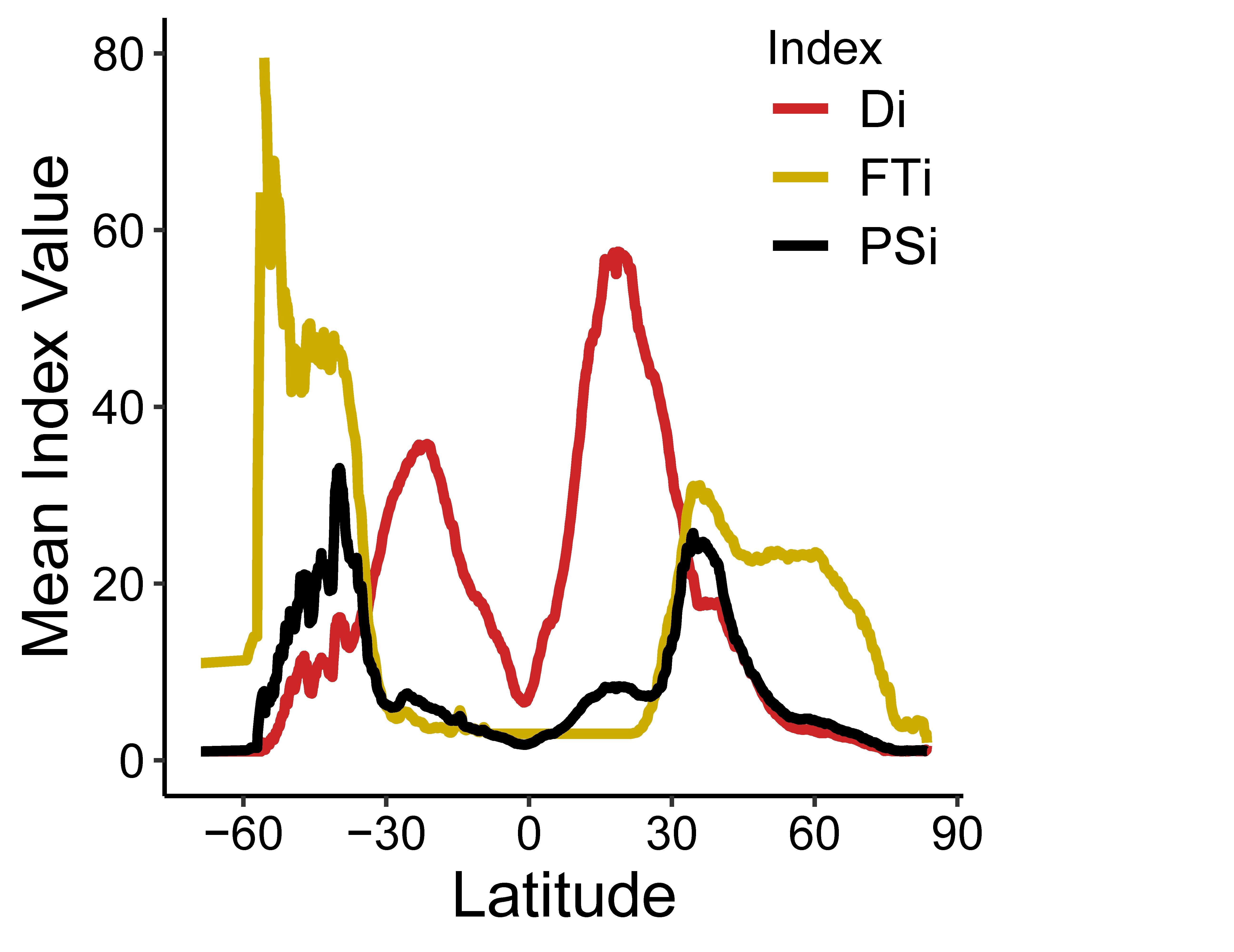

Supplement: fig_S4_tpac102 [file fig_s4_tpac102.jpeg]
