# Supplementary material for: At least it is a dry cold: the global distribution of freeze–thaw and drought stress and the traits that may impart poly-tolerance in conifers
Source: Tree Physiol. 2022 Sep 12;43(1):1–15. doi: 10.1093/treephys/tpac102 (PMC9833871; doi:10.1093/treephys/tpac102)
Supplement: Supplemental_Table1_tpac102 [file supplemental_table1_tpac102.pdf]

Supplemental Table S1: All conifer species within our dataset. “Occur coverage” is the the total FT-D space each species occupies as a percent of the total FT-D space occupied by conifers (2700 index units^2). We quantified this space with a buffer around each point, as shown in Fig. 3. “Trait coverage” is the percent of the occurrence area where traits have been measured. “Realm” shows up to three realms where the species occurs most often. We included realms represented by at least 10 points or 5%. Species are grouped by their dominant realm and then sorted by decreasing trait coverage.

Species	Occur coverage (%)	Trait coverage (%)	Realm
Afrocarpus dawei	3.1	0	Afrotropic
Afrocarpus falcatus	4.3	0	—
Afrocarpus gracilior	7.5	0	—
Afrocarpus usambarensis	3.4	0	—
Juniperus procera	10.4	0	—
Podocarpus capuronii	1.6	0	—
Podocarpus elongatus	5.8	0	— ; Australasia
Podocarpus henkelii	8.3	0	— ; Australasia
Podocarpus humbertii	1.5	0	—
Podocarpus latifolius	7.1	0	— ; Australasia
Podocarpus madagascariensis	3.0	0	—
Podocarpus milanjianus	6.3	0	—
Podocarpus perrieri	1.6	0	—
Podocarpus rostratus	1.1	0	—
Widdringtonia nodiflora	3.4	0	—
Widdringtonia schwarzii	6.1	0	— ; Australasia
Widdringtonia wallichii	2.9	0	—
Widdringtonia whytei	1.3	0	— ; Australasia
Retrophyllum minus	1.3	58.3	Australasia
Araucaria laubenfelsii	1.5	51.2	—
Agathis ovata	1.8	43.8	—
Falcatifolium taxoides	1.8	43.8	—
Parasitaxus usta	2.0	39.6	—
Callitris neocaledonica	2.0	38.9	—
Callitris pancheri	2.0	38.9	—
Callitris sulcata	2.9	27.3	—
Callitris acuminata	4.6	16.9	—
Callitris drummondii	5.9	13.1	—
Dacrycarpus imbricatus	5.9	13.1	— ; Indomalayan; Oceania
Podocarpus laetus	11.1	13.0	—
Pectinopitys ferruginea	10.6	12.5	—
Pinus pinea	28.7	12.3	— ; Palearctic
Dacrydium cupressinum	15.8	11.5	—
Agathis australis	11.8	11.3	—
Callitris arenaria	7.6	10.2	—
Phyllocladus trichomanoides	13.1	10.2	—
Halocarpus kirkii	7.7	8.7	—
Araucaria columnaris	9.4	8.3	—
Callitris roei	9.4	8.3	—
Pinus elliottii	17.7	8.1	— ; Nearctic
Pinus halepensis	44.3	7.0	— ; Palearctic
Pinus pinaster	33.5	5.9	— ; Palearctic
Callitris canescens	15.1	5.2	—
Callitris muelleri	15.1	5.1	—
Dacrycarpus dacrydioides	15.0	4.4	—
Platycladus orientalis	20.4	3.8	— ; Palearctic; Nearctic
Cupressus torulosa	21.8	3.6	— ; Palearctic; Indomalayan

continued

Species	Occur coverage (%)	Trait coverage (%)	Realm	
Cryptomeria japonica	18.9	3.5	—	; Palearctic; Indomalayan
Callitris verrucosa	22.1	3.5	—	
Callitris preissii	28.0	2.8	—	
Callitris columellaris	42.3	1.8	—	
Abies numidica	2.5	0	—	; Palearctic
Acmopyle pancheri	1.7	0	—	
Agathis atropurpurea	2.7	0	—	
Agathis dammara	3.1	0	—	; Indomalayan
Agathis labillardierei	0.7	0	—	
Agathis lanceolata	1.7	0	—	
Agathis microstachya	2.1	0	—	
Agathis montana	0.8	0	—	
Agathis moorei	5.1	0	—	
Agathis robusta	12.0	0	—	
Agathis silbae	0.5	0	—	
Araucaria bernieri	1.4	0	—	
Araucaria bidwillii	27.4	0	—	
Araucaria biramulata	2.1	0	—	
Araucaria cunninghamii	17.5	0	—	
Araucaria heterophylla	22.5	0	—	
Araucaria humboldtensis	1.4	0	—	
Araucaria hunsteinii	2.9	0	—	
Araucaria luxurians	1.9	0	—	
Araucaria montana	2.4	0	—	
Araucaria muelleri	1.6	0	—	
Araucaria nemorosa	0.8	0	—	
Araucaria rulei	2.4	0	—	
Araucaria schmidii	0.8	0	—	
Araucaria scopulorum	1.9	0	—	
Araucaria subulata	1.3	0	—	
Athrotaxis cupressoides	18.7	0	—	
Athrotaxis selaginoides	13.2	0	—	
Austrotaxus spicata	1.7	0	—	
Callitris baileyi	4.7	0	—	
Callitris endlicheri	49.0	0	—	
Callitris gracilis	27.6	0	—	
Callitris macleayana	11.1	0	—	
Callitris monticola	4.7	0	—	
Callitris oblonga	16.6	0	—	
Callitris pyramidalis	12.6	0	—	
Callitris rhomboidea	44.4	0	—	
Cedrus atlantica	20.6	0	—	; Palearctic
Cedrus deodara	27.8	0	—	; Indomalayan; Palearctic
Chamaecyparis lawsoniana	22.1	0	—	; Nearctic
Cupressus sempervirens	36.7	0	—	; Palearctic
Dacrycarpus cinctus	0.9	0	—	
Dacrycarpus compactus	0.7	0	—	
Dacrycarpus expansus	0.9	0	—	
Dacrycarpus steupii	0.9	0	—	; Indomalayan
Dacrycarpus vieillardii	2.0	0	—	
Dacrydium araucarioides	1.3	0	—	
Dacrydium balansae	1.7	0	—	
Dacrydium cornwallianum	0.5	0	—	

continued

Species	Occur coverage (%)	Trait coverage (%)	Realm	
Dacrydium guillauminii	0.8	0	—	
Dacrydium leptophyllum	0.5	0	—	
Dacrydium lycopodioides	1.3	0	—	
Dacrydium magnum	0.9	0	—	
Dacrydium nidulum	1.8	0	—	; Oceania
Dacrydium novoguineense	1.0	0	—	
Dacrydium spathoides	0.5	0	—	
Diselma archeri	4.9	0	—	
Falcatifolium papuanum	1.0	0	—	
Falcatifolium sleumeri	0.5	0	—	
Halocarpus bidwillii	7.5	0	—	
Halocarpus biformis	9.9	0	—	
Hesperocyparis arizonica	40.3	0	—	; Nearctic
Hesperocyparis glabra	7.4	0	—	
Hesperocyparis lusitanica	28.5	0	—	; Neotropic; Nearctic
Hesperocyparis macrocarpa	19.4	0	—	
Juniperus bermudiana	6.5	0	—	
Juniperus taxifolia	1.7	0	—	; Indomalayan; Palearctic
Lagarostrobos franklinii	9.3	0	—	
Lepidothamnus intermedius	10.3	0	—	
Lepidothamnus laxifolius	9.6	0	—	
Libocedrus austrocaledonica	1.4	0	—	
Libocedrus bidwillii	7.9	0	—	
Libocedrus chevalieri	0.9	0	—	
Libocedrus plumosa	13.6	0	—	
Libocedrus yateensis	1.0	0	—	
Manoao colensoi	11.4	0	—	
Metasequoia glyptostroboides	9.0	0	—	; Palearctic; Nearctic
Microcachrys tetragona	4.7	0	—	
Papuacedrus papuana	1.4	0	—	
Pectinopitys ferruginoides	1.6	0	—	
Pectinopitys ladei	1.1	0	—	
Pherosphaera fitzgeraldii	3.9	0	—	
Pherosphaera hookeriana	4.8	0	—	
Phyllocladus aspleniifolius	14.0	0	—	
Phyllocladus hypophyllus	2.0	0	—	; Indomalayan
Phyllocladus toatoa	8.9	0	—	
Picea smithiana	15.3	0	—	; Indomalayan; Palearctic
Pinus canariensis	18.2	0	—	; Palearctic
Pinus muricata	16.4	0	—	; Nearctic
Pinus patula	25.6	0	—	; Neotropic; Nearctic
Pinus radiata	53.4	0	—	
Pinus torreyana	5.4	0	—	; Nearctic
Podocarpus acutifolius	5.7	0	—	
Podocarpus archboldii	1.4	0	—	
Podocarpus brassii	0.7	0	—	
Podocarpus decumbens	1.0	0	—	
Podocarpus dispermus	1.9	0	—	
Podocarpus drouynianus	6.1	0	—	
Podocarpus elatus	23.7	0	—	
Podocarpus glaucus	1.0	0	—	
Podocarpus gnidioides	1.4	0	—	
Podocarpus grayae	8.2	0	—	

continued

Species	Occur coverage (%)	Trait coverage (%)	Realm	
Podocarpus insularis	0.9	0	—	
Podocarpus ledermannii	1.6	0	—	
Podocarpus levis	1.4	0	—	; Indomalayan
Podocarpus longifoliolatus	1.3	0	—	
Podocarpus lucienii	1.6	0	—	
Podocarpus nivalis	8.8	0	—	
Podocarpus novae-caledoniae	2.4	0	—	
Podocarpus orarius	1.4	0	—	
Podocarpus pilgeri	7.0	0	—	; Indomalayan
Podocarpus polyspermus	1.5	0	—	
Podocarpus pseudobracteatus	0.9	0	—	
Podocarpus rubens	1.2	0	—	; Indomalayan
Podocarpus rumphii	3.7	0	—	; Indomalayan
Podocarpus salomoniensis	0.5	0	—	
Podocarpus smithii	2.1	0	—	
Podocarpus spinulosus	32.1	0	—	
Podocarpus sylvestris	1.7	0	—	
Podocarpus totara	15.0	0	—	
Prumnopitys taxifolia	14.0	0	—	
Retrophyllum comptonii	1.4	0	—	
Retrophyllum vitiense	0.9	0	—	; Oceania
Sundacarpus amarus	3.4	0	—	; Indomalayan
Wollemia nobilis	2.1	0	—	
Pinus krempfii	1.1	67.7	Indomalayan	
Pinus dalatensis	1.5	51.2	—	
Agathis borneensis	1.8	27.1	—	
Nageia wallichiana	4.2	18.6	—	; Australasia
Dacrydium elatum	4.4	17.8	—	
Podocarpus neriifolius	14.6	10.7	—	; Australasia; Palearctic
Pinus kesiya	7.8	10.0	—	; Australasia
Nageia nagi	12.0	6.5	—	; Palearctic
Fokienia hodginsii	16.0	4.8	—	; Palearctic; Australasia
Pinus massoniana	16.3	4.8	—	; Palearctic; Australasia
Taxus wallichiana	16.8	4.6	—	; Palearctic; Australasia
Pinus roxburghii	20.9	3.7	—	; Australasia; Palearctic
Abies beshanzuensis	0.5	0	—	
Abies fanjingshanensis	1.0	0	—	
Abies kawakamii	1.4	0	—	
Abies pindrow	16.6	0	—	; Australasia; Palearctic
Abies spectabilis	13.6	0	—	; Palearctic; Australasia
Abies yuanbaoshanensis	0.8	0	—	
Abies ziyuanensis	1.1	0	—	
Agathis flavescens	0.7	0	—	
Agathis kinabaluensis	0.7	0	—	
Agathis lenticula	0.7	0	—	
Agathis orbicula	0.5	0	—	
Amentotaxus argotaenia	13.5	0	—	; Palearctic
Amentotaxus assamica	2.1	0	—	
Amentotaxus formosana	1.5	0	—	
Amentotaxus hatuyenensis	0.8	0	—	
Amentotaxus poilanei	0.8	0	—	
Amentotaxus yunnanensis	5.2	0	—	; Palearctic
Calocedrus formosana	1.4	0	—	

continued

Species	Occur coverage (%)	Trait coverage (%)	Realm	
Calocedrus macrolepis	7.2	0	—	; Palearctic
Calocedrus rupestris	4.3	0	—	
Cathaya argyrophylla	2.9	0	—	; Palearctic
Cephalotaxus hainanensis	1.0	0	—	
Cephalotaxus mannii	10.3	0	—	; Palearctic
Chamaecyparis formosensis	3.1	0	—	; Australasia
Cunninghamia konishii	6.2	0	—	; Australasia
Cupressus cashmeriana	9.4	0	—	; Australasia
Dacrycarpus cumingii	2.0	0	—	
Dacrycarpus kinabaluensis	0.7	0	—	
Dacrydium beccarii	1.6	0	—	; Australasia
Dacrydium comosum	0.7	0	—	
Dacrydium ericoides	0.5	0	—	
Dacrydium gibbsiae	0.7	0	—	
Dacrydium gracile	0.7	0	—	
Dacrydium medium	0.9	0	—	
Dacrydium pectinatum	2.7	0	—	
Dacrydium xanthandrum	0.9	0	—	; Australasia
Falcatifolium angustum	0.5	0	—	
Falcatifolium falciforme	1.3	0	—	
Falcatifolium gruezoi	2.0	0	—	; Australasia
Glyptostrobus pensilis	8.1	0	—	
Keteleeria fortunei	13.4	0	—	; Australasia; Palearctic
Nageia fleuryi	4.6	0	—	
Nageia maxima	0.5	0	—	
Nageia motleyi	1.8	0	—	
Nothotsuga longibracteata	8.0	0	—	
Picea morrisonicola	1.4	0	—	; Australasia
Picea spinulosa	6.3	0	—	; Palearctic; Australasia
Pinus amamiana	0.7	0	—	
Pinus bhutanica	6.1	0	—	; Palearctic
Pinus fenzeliana	10.7	0	—	
Pinus hwangshanensis	8.5	0	—	; Palearctic; Australasia
Pinus latteri	4.1	0	—	
Pinus luchuensis	3.9	0	—	; Australasia
Pinus merkusii	3.1	0	—	
Pinus morrisonicola	3.4	0	—	; Australasia
Pinus taiwanensis	3.4	0	—	; Australasia
Pinus wallichiana	22.5	0	—	; Palearctic; Australasia
Pinus wangii	1.9	0	—	; Palearctic
Podocarpus atjehensis	1.1	0	—	
Podocarpus borneensis	0.5	0	—	
Podocarpus bracteatus	2.5	0	—	; Australasia
Podocarpus brevifolius	0.5	0	—	
Podocarpus chingianus	0.8	0	—	
Podocarpus confertus	0.5	0	—	
Podocarpus costalis	0.9	0	—	
Podocarpus deflexus	0.7	0	—	
Podocarpus fasciculus	1.9	0	—	
Podocarpus gibbsiae	0.7	0	—	
Podocarpus globulus	0.5	0	—	
Podocarpus laubenfelsii	0.7	0	—	
Podocarpus lophatus	1.6	0	—	

continued

Species	Occur coverage (%)	Trait coverage (%)	Realm
Podocarpus macrocarpus	3.1	0	—
Podocarpus micropedunculatus	0.5	0	—
Podocarpus nakaii	2.0	0	—
Podocarpus palawanensis	0.8	0	—
Podocarpus polystachyus	2.3	0	— ; Australasia
Podocarpus ramosii	1.2	0	—
Podocarpus ridleyi	1.4	0	—
Podocarpus spathoides	1.0	0	—
Podocarpus teysmannii	1.4	0	—
Pseudotaxus chienii	5.4	0	— ; Palearctic
Taiwania cryptomerioides	8.0	0	— ; Palearctic; Australasia
Taxus contorta	14.4	0	— ; Palearctic
Torreya jackii	2.4	0	— ; Palearctic
Xanthocyparis vietnamensis	2.0	0	—
Abies fraseri	2.3	29.5	Nearctic
Picea chihuahuana	2.7	28.8	—
Larix occidentalis	9.2	26.5	—
Picea mariana	9.0	23.8	—
Abies lasiocarpa	17.2	22.4	—
Picea laxa	7.7	22.0	—
Juniperus flaccida	7.9	19.8	— ; Neotropic
Sequoia sempervirens	22.9	18.9	— ; Australasia
Juniperus monticola	4.1	18.9	— ; Neotropic
Juniperus scopulorum	20.1	18.8	—
Pinus contorta	29.0	18.0	— ; Australasia
Pseudotsuga menziesii	43.9	17.1	— ; Australasia
Tsuga heterophylla	16.0	16.9	— ; Australasia
Pinus virginiana	10.0	15.6	— ; Australasia
Pinus banksiana	10.9	13.9	— ; Australasia
Pinus ponderosa	38.9	13.6	— ; Australasia
Pinus resinosa	7.6	13.6	— ; Australasia
Pinus palustris	17.8	13.1	— ; Australasia
Pinus albicaulis	12.1	12.8	—
Juniperus monosperma	20.0	12.6	—
Larix laricina	6.2	12.5	—
Larix lyallii	6.3	12.3	—
Abies balsamea	6.4	12.1	—
Picea engelmannii	19.7	11.8	—
Pinus edulis	23.0	11.6	—
Picea rubens	6.1	10.9	—
Juniperus ashei	11.1	10.7	—
Thuja occidentalis	11.4	10.4	— ; Australasia
Juniperus osteosperma	23.6	9.9	—
Pinus strobus	17.5	8.3	— ; Neotropic; Australasia
Juniperus deppeana	19.6	8.0	— ; Neotropic
Pinus taeda	19.0	7.4	— ; Australasia
Tsuga canadensis	9.1	7.3	— ; Australasia
Abies grandis	16.1	7.1	— ; Australasia
Taxodium distichum	21.9	7.1	— ; Australasia; Neotropic
Abies amabilis	11.2	6.9	— ; Australasia
Pinus rigida	9.6	6.9	— ; Australasia
Juniperus virginiana	23.6	6.6	— ; Australasia
Picea sitchensis	11.8	6.6	— ; Australasia

continued

Species	Occur coverage (%)	Trait coverage (%)	Realm
Thuja plicata	19.1	6.0	— ; Australasia
Juniperus arizonica	13.2	5.9	—
Pinus arizonica	13.3	5.8	—
Pinus serotina	13.4	5.8	— ; Australasia
Pinus echinata	14.2	5.5	— ; Australasia
Juniperus pinchotii	15.3	5.1	—
Juniperus occidentalis	16.4	4.8	—
Pinus monticola	16.8	4.6	— ; Australasia
Pinus leiophylla	17.2	4.5	— ; Neotropic
Pinus strobiformis	17.3	4.5	— ; Neotropic
Pinus cembroides	21.6	3.6	— ; Neotropic
Juniperus californica	24.2	3.2	—
Pinus flexilis	28.9	2.7	— ; Australasia
Abies concolor	32.8	2.4	— ; Australasia
Pinus monophylla	37.3	2.1	—
Abies bracteata	5.8	0	— ; Australasia
Abies durangensis	5.8	0	—
Abies hidalgensis	1.0	0	— ; Neotropic
Abies magnifica	13.7	0	— ; Australasia
Abies procera	10.0	0	— ; Australasia
Abies vejarii	4.6	0	— ; Australasia
Callitropsis nootkatensis	8.3	0	—
Calocedrus decurrens	26.9	0	— ; Australasia
Chamaecyparis thyoides	11.3	0	— ; Australasia
Hesperocyparis bakeri	2.6	0	—
Hesperocyparis goveniana	10.3	0	— ; Australasia
Hesperocyparis guadalupensis	3.4	0	—
Hesperocyparis macnabiana	6.6	0	—
Hesperocyparis sargentii	12.6	0	—
Juniperus angosturana	3.3	0	—
Juniperus blancoi	5.5	0	— ; Neotropic
Juniperus coahuilensis	7.0	0	—
Juniperus durangensis	6.9	0	—
Juniperus horizontalis	14.3	0	—
Juniperus procumbens	3.6	0	— ; Australasia; Indomalayan
Juniperus saltillensis	4.7	0	—
Picea breweriana	5.3	0	—
Picea martinezii	2.0	0	—
Picea pungens	15.7	0	— ; Australasia
Pinus aristata	8.8	0	— ; Australasia
Pinus attenuata	25.0	0	— ; Australasia
Pinus balfouriana	4.7	0	—
Pinus clausa	7.0	0	— ; Australasia
Pinus coulteri	17.5	0	— ; Australasia
Pinus culminicola	2.6	0	—
Pinus durangensis	12.1	0	— ; Neotropic; Australasia
Pinus engelmannii	17.1	0	— ; Australasia
Pinus glabra	7.8	0	—
Pinus greggii	7.0	0	— ; Neotropic; Australasia
Pinus jeffreyi	25.4	0	— ; Australasia
Pinus lambertiana	24.3	0	— ; Australasia
Pinus longaeva	11.3	0	—
Pinus lumholtzii	6.6	0	— ; Neotropic

continued

Species	Occur coverage (%)	Trait coverage (%)	Realm
Pinus maximartinezii	2.2	0	— ; Neotropic
Pinus nelsonii	2.8	0	—
Pinus pinceana	3.9	0	—
Pinus pungens	5.3	0	—
Pinus quadrifolia	6.0	0	—
Pinus remota	7.9	0	—
Pinus sabiniana	25.1	0	— ; Australasia
Pseudotsuga macrocarpa	7.1	0	—
Sequoiadendron giganteum	19.9	0	— ; Australasia
Taxus brevifolia	15.8	0	—
Taxus canadensis	5.8	0	—
Taxus floridana	1.0	0	—
Torreya californica	17.3	0	— ; Australasia
Torreya taxifolia	1.1	0	—
Tsuga caroliniana	2.7	0	—
Tsuga mertensiana	11.0	0	—
Juniperus barbadensis	3.4	35.5	Neotropic
Podocarpus salicifolius	2.9	26.9	—
Podocarpus nubigenus	8.1	19.1	—
Pinus caribaea	6.1	12.8	— ; Australasia
Prumnopitys andina	6.3	12.4	—
Pinus hartwegii	8.6	9.1	— ; Nearctic; Australasia
Pinus pseudostrobus	9.8	7.9	— ; Nearctic; Australasia
Fitzroya cupressoides	9.1	7.3	—
Pinus devoniana	10.7	7.2	— ; Nearctic; Australasia
Saxegothaea conspicua	12.6	6.2	—
Podocarpus salignus	13.6	5.7	— ; Australasia
Austrocedrus chilensis	15.3	5.1	—
Abies guatemalensis	4.1	0	—
Abies hickelii	3.8	0	—
Abies religiosa	6.9	0	— ; Nearctic; Australasia
Araucaria angustifolia	7.4	0	— ; Australasia
Araucaria araucana	11.5	0	— ; Australasia
Juniperus comitana	1.5	0	—
Juniperus gamboana	3.6	0	—
Juniperus gracilior	3.2	0	—
Juniperus jaliscana	1.1	0	—
Juniperus saxicola	0.8	0	—
Juniperus standleyi	1.7	0	—
Lepidothamnus fonkii	5.3	0	—
Pectinopitys exigua	2.2	0	—
Pectinopitys harmsiana	4.9	0	—
Pectinopitys standleyi	0.9	0	—
Pilgerodendron uviferum	7.3	0	—
Pinus ayacahuite	10.5	0	— ; Australasia; Nearctic
Pinus cubensis	3.1	0	—
Pinus douglasiana	5.1	0	— ; Nearctic
Pinus herrerae	6.1	0	— ; Nearctic
Pinus jaliscana	2.6	0	—
Pinus lawsonii	7.1	0	— ; Australasia
Pinus luzmariae	3.7	0	— ; Nearctic
Pinus maximinoi	8.2	0	—
Pinus montezumae	11.8	0	— ; Nearctic; Australasia

continued

Species	Occur coverage (%)	Trait coverage (%)	Realm
Pinus occidentalis	5.1	0	—
Pinus oocarpa	9.6	0	— ; Nearctic
Pinus praetermissa	4.8	0	— ; Nearctic
Pinus pringlei	5.9	0	—
Pinus rzedowskii	1.1	0	—
Pinus tecunumanii	5.4	0	—
Pinus teocote	10.2	0	— ; Nearctic
Pinus tropicalis	1.3	0	—
Podocarpus acuminatus	1.3	0	—
Podocarpus angustifolius	2.3	0	—
Podocarpus aracensis	1.6	0	—
Podocarpus brasiliensis	2.8	0	—
Podocarpus buchii	2.6	0	—
Podocarpus celatus	3.0	0	—
Podocarpus coriaceus	1.8	0	—
Podocarpus costaricensis	1.1	0	—
Podocarpus ekmanii	1.9	0	—
Podocarpus glomeratus	5.4	0	—
Podocarpus guatemalensis	3.8	0	—
Podocarpus hispaniolensis	1.1	0	—
Podocarpus lambertii	4.7	0	—
Podocarpus magnifolius	3.6	0	—
Podocarpus matudae	5.3	0	—
Podocarpus oleifolius	4.4	0	—
Podocarpus parlatorei	7.2	0	—
Podocarpus pendulifolius	1.5	0	—
Podocarpus purdieanus	1.0	0	—
Podocarpus roraimae	1.4	0	—
Podocarpus rusbyi	2.7	0	—
Podocarpus sellowii	1.8	0	—
Podocarpus sprucei	1.9	0	—
Podocarpus steyermarkii	2.3	0	—
Podocarpus tepuiensis	2.1	0	—
Podocarpus transiens	2.3	0	—
Podocarpus trinitensis	1.1	0	—
Podocarpus urbanii	0.9	0	—
Prumnopitys montana	3.9	0	—
Retrophyllum piresii	0.8	0	—
Retrophyllum rospigliosii	4.3	0	—
Taxodium distichum var. *mexicanum*	7.6	0	— ; Nearctic
Taxus globosa	4.9	0	— ; Nearctic
Acmopyle sahniana	0.5	0	Oceania
Agathis macrophylla	4.5	0	— ; Australasia
Dacrydium nausoriense	1.2	0	—
Podocarpus affinis	0.5	0	—
Podocarpus pallidus	0.5	0	—
Pinus koraiensis	5.2	52.5	Palearctic; Australasia
Larix decidua	9.3	36.4	— ; Australasia
Picea abies	14.4	31.7	— ; Australasia
Picea koraiensis	2.5	31.3	—
Pinus sylvestris	23.8	29.9	— ; Australasia
Abies alba	8.6	24.9	— ; Australasia
Larix gmelinii	10.3	22.7	—

continued

Species	Occur coverage (%)	Trait coverage (%)	Realm	
Abies nephrolepis	4.1	18.8	—	
Abies holophylla	4.4	17.6	—	; Australasia
Pinus densiflora	11.3	15.0	—	; Australasia
Pinus sibirica	6.3	12.4	—	
Abies mariesii	4.3	11.3	—	; Australasia
Pinus cembra	4.6	10.4	—	; Australasia
Larix kaempferi	7.7	10.1	—	; Australasia
Picea obovata	7.9	9.9	—	
Pinus thunbergii	8.0	9.8	—	; Australasia
Pinus nigra	28.6	9.6	—	; Australasia
Abies firma	7.2	9.2	—	; Australasia
Pinus pumila	5.3	9.2	—	
Abies sibirica	9.4	8.3	—	
Abies fargesii	9.5	8.2	—	; Australasia
Pinus tabuliformis	19.4	8.0	—	; Australasia
Taxus baccata	27.7	7.5	—	; Australasia
Keteleeria evelyniana	10.8	7.2	—	; Indomalayan
Larix sibirica	10.9	7.1	—	
Chamaecyparis obtusa	12.0	6.5	—	; Australasia; Indomalayan
Tetraclinis articulata	13.9	5.6	—	
Juniperus communis	31.1	5.4	—	; Nearctic
Pinus mugo	10.1	4.7	—	; Australasia
Cedrus libani	16.8	4.6	—	; Australasia
Juniperus thurifera	17.5	4.4	—	
Juniperus chinensis	24.4	3.2	—	; Australasia; Indomalayan
Juniperus oxycedrus	30.7	2.5	—	
Abies cephalonica	9.0	0	—	; Australasia
Abies chensiensis	12.4	0	—	
Abies cilicica	9.0	0	—	
Abies delavayi	12.1	0	—	; Indomalayan; Australasia
Abies densa	9.3	0	—	; Indomalayan
Abies fabri	4.6	0	—	
Abies forrestii	13.9	0	—	
Abies homolepis	5.1	0	—	; Australasia
Abies koreana	2.5	0	—	; Australasia
Abies nebrodensis	2.1	0	—	; Australasia
Abies nordmanniana	13.1	0	—	; Australasia
Abies pinsapo	8.1	0	—	; Australasia
Abies recurvata	9.4	0	—	; Australasia
Abies sachalinensis	1.9	0	—	
Abies squamata	8.2	0	—	
Abies veitchii	4.4	0	—	; Australasia
Cephalotaxus fortunei	19.6	0	—	; Indomalayan
Cephalotaxus griffithii	0.8	0	—	
Cephalotaxus harringtonia	19.1	0	—	; Indomalayan; Australasia
Cephalotaxus nana	2.5	0	—	; Indomalayan
Cephalotaxus oliveri	5.1	0	—	; Indomalayan
Chamaecyparis pisifera	11.5	0	—	; Australasia
Cunninghamia lanceolata	20.0	0	—	; Indomalayan; Australasia
Cupressus chengiana	6.6	0	—	
Cupressus duclouxiana	13.3	0	—	; Australasia
Cupressus dupreziana	3.8	0	—	
Cupressus pendula	7.3	0	—	; Indomalayan

continued

Species	Occur coverage (%)	Trait coverage (%)	Realm	
Juniperus brevifolia	1.9	0	—	
Juniperus cedrus	2.2	0	—	
Juniperus chinensis var. *chinensis*	2.3	0	—	
Juniperus convallium	12.9	0	—	
Juniperus drupacea	10.4	0	—	
Juniperus excelsa	38.2	0	—	
Juniperus foetidissima	16.1	0	—	
Juniperus formosana	19.3	0	—	; Indomalayan
Juniperus indica	17.9	0	—	; Indomalayan
Juniperus komarovii	7.4	0	—	
Juniperus phoenicea	33.8	0	—	
Juniperus pingii	19.0	0	—	
Juniperus przewalskii	11.8	0	—	
Juniperus pseudosabina	14.1	0	—	
Juniperus recurva	22.3	0	—	; Indomalayan
Juniperus rigida	14.1	0	—	; Australasia
Juniperus sabina	24.5	0	—	
Juniperus saltuaria	9.5	0	—	
Juniperus semiglobosa	13.8	0	—	; Indomalayan
Juniperus squamata	26.8	0	—	; Indomalayan
Juniperus tibetica	14.8	0	—	
Keteleeria davidiana	15.1	0	—	; Indomalayan
Larix griffithii	13.3	0	—	; Indomalayan
Larix mastersiana	3.4	0	—	
Larix potaninii	15.7	0	—	
Microbiota decussata	1.7	0	—	
Picea alcoquiana	2.1	0	—	; Australasia
Picea asperata	11.7	0	—	; Australasia
Picea aurantiaca	1.7	0	—	
Picea brachytyla	12.0	0	—	
Picea crassifolia	13.4	0	—	
Picea farreri	2.5	0	—	
Picea glehnii	3.8	0	—	; Australasia
Picea jezoensis	5.5	0	—	
Picea koyamae	2.3	0	—	; Australasia
Picea likiangensis	13.0	0	—	; Australasia
Picea linzhiensis	6.0	0	—	
Picea maximowiczii	0.6	0	—	
Picea meyeri	6.5	0	—	
Picea neoveitchii	4.3	0	—	
Picea omorika	4.3	0	—	; Australasia
Picea orientalis	10.1	0	—	; Australasia
Picea polita	2.1	0	—	
Picea purpurea	9.0	0	—	
Picea retroflexa	7.5	0	—	; Australasia
Picea schrenkiana	10.8	0	—	
Picea wilsonii	12.0	0	—	
Pinus brutia	27.4	0	—	; Australasia
Pinus bungeana	12.9	0	—	; Australasia
Pinus densata	12.2	0	—	; Australasia
Pinus gerardiana	14.7	0	—	; Indomalayan; Australasia
Pinus heldreichii	10.8	0	—	; Australasia
Pinus henryi	4.6	0	—	

continued

Species	Occur coverage (%)	Trait coverage (%)	Realm	
Pinus parviflora	5.3	0	—	; Australasia
Pinus peuce	4.7	0	—	
Pinus squamata	0.8	0	—	
Pinus uncinata	4.2	0	—	
Pinus yunnanensis	20.4	0	—	; Indomalayan; Australasia
Podocarpus macrophyllus	16.1	0	—	; Indomalayan; Australasia
Podocarpus subtropicalis	1.0	0	—	
Pseudolarix amabilis	4.7	0	—	; Australasia; Indomalayan
Pseudotsuga japonica	0.9	0	—	
Pseudotsuga sinensis	13.8	0	—	; Indomalayan
Sciadopitys verticillata	2.9	0	—	
Taxus chinensis	10.2	0	—	; Indomalayan
Taxus cuspidata	4.0	0	—	
Taxus mairei	11.7	0	—	; Indomalayan
Thuja koraiensis	2.1	0	—	
Thuja standishii	2.8	0	—	
Thuja sutchuenensis	0.9	0	—	
Thujopsis dolabrata	10.3	0	—	; Australasia
Torreya fargesii	8.0	0	—	
Torreya grandis	4.4	0	—	; Indomalayan
Torreya nucifera	3.9	0	—	
Tsuga chinensis	20.1	0	—	; Indomalayan
Tsuga diversifolia	2.2	0	—	
Tsuga dumosa	24.7	0	—	; Indomalayan
Tsuga forrestii	9.1	0	—	
Tsuga sieboldii	3.5	0	—	; Australasia
