# Supplementary material for: At least it is a dry cold: the global distribution of freeze–thaw and drought stress and the traits that may impart poly-tolerance in conifers
Source: Tree Physiol. 2022 Sep 12;43(1):1–15. doi: 10.1093/treephys/tpac102 (PMC9833871; doi:10.1093/treephys/tpac102)

**Supplementary Table S2.** Regression analysis summary of the effect of Di, FTi, PSi, Families, and their interaction on LMA, TLP, and P50.


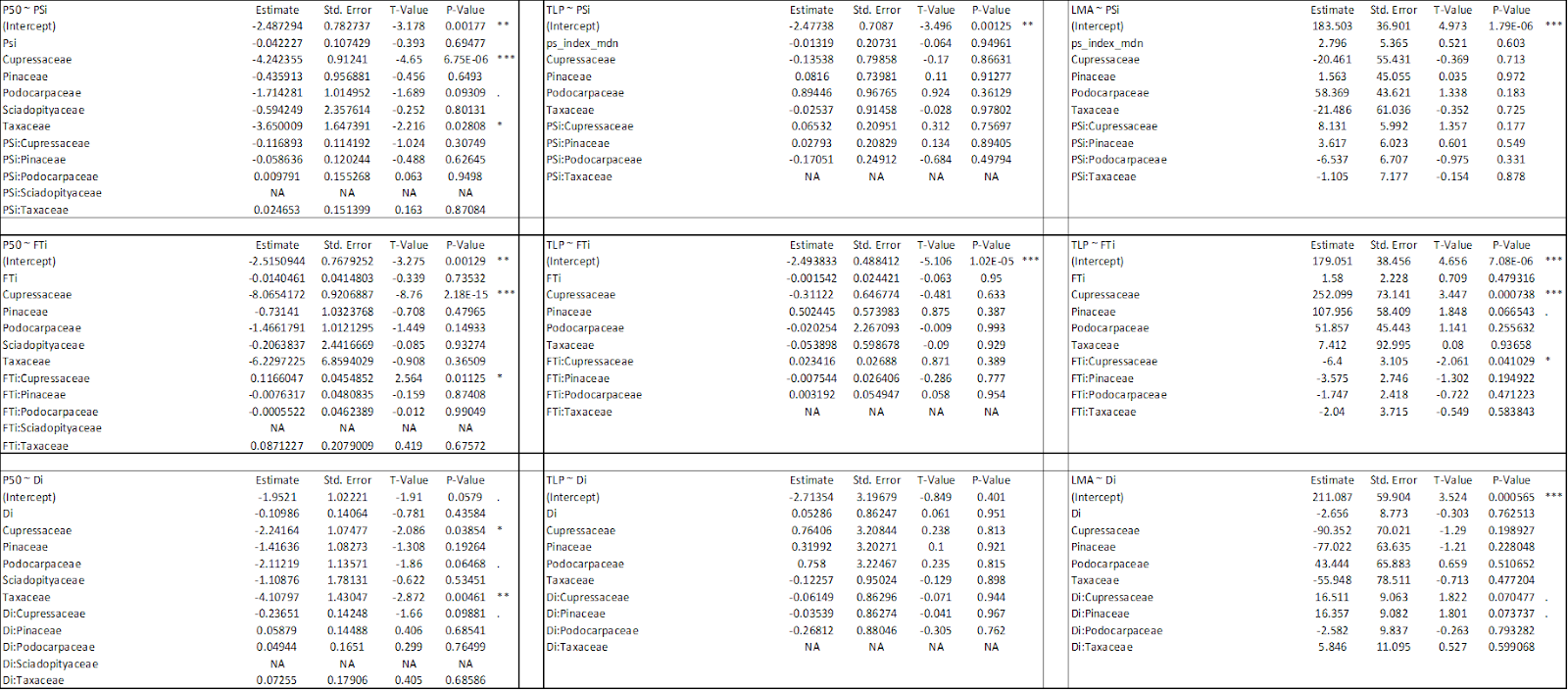

Supplement: Supplementary_Table_S2_tpac102 [file supplementary_table_s2_tpac102.docx]
