# Supplementary material for: At least it is a dry cold: the global distribution of freeze–thaw and drought stress and the traits that may impart poly-tolerance in conifers
Source: Tree Physiol. 2022 Sep 12;43(1):1–15. doi: 10.1093/treephys/tpac102 (PMC9833871; doi:10.1093/treephys/tpac102)
Supplement: Supplemental_materials_final_tpac102 [file supplemental_materials_final_tpac102.docx]

# **Supplementary Methods**

*Species taxonomy.*

We followed the taxonomic authority of Conifers of the World and the KEW World Checklist of Vascular Plant Species (Farjon et al. 2018; Govaerts et al. 2021). Of the 699 species described in this list, were were able to find occurence and environmental data for 604 species (see below) and trait data for 210 (P50), 202 (LMA), and 52 (TLP) species (see below).

*Identifying traits that impart tolerance to drought, FT cycles, and their overlap.*

Trait data for conifers were gathered from 173 different publications. We collected the stem P_50_ for 210 species from 95 different papers. For stem P_50_, we excluded data that were generated from freeze-thaw curves (e.g., Pittermann & Sperry 2006; Willson & Jackson 2006), artificially changed the surface tension or osmolality of the water to encourage a response (Losso et al. 2017), or were measured after inducing pit fatigue (Feng et al. 2021). We also excluded data that measured seedlings if they did not have branches and ‘stems’ were considered the main trunk of the seedling (e.g., Kavanagh et al. 1999; Kavanagh & Zaerr 1997; see Domec & Gartner 2002 for comparison). Some stem P_50_ were extracted from figures of certain papers (e.g., Charra-Vaskou et al. 2012). The Xylem Functional Trait Database (Choat et al. 2012) was used to corroborate the extracted stem P_50_ when available (e.g., Cochard 1992; Piñol & Sala 2000), as well as values that were not present in the main text or figures of a single paper (Brodribb & Cochard 2009).

We collected the leaf mass per area (LMA) for 202 species from 72 different papers. We excluded LMA measured on cotyledons and primary needles/leaves, which are substantially lower than LMA values for mature needles/leaves (see Kuusk et al. 2018 or Mediavilla et al. 2014 for a comparison). Some species are overly represented in the seedling literature, which decreased their mean LMA substantially relative to species that only have data on mature needles/leaves. Not all papers report whether the needles/leaves were primary or mature, so we excluded LMA that were referred to as seedlings or were individuals that were less than one year old. We also excluded papers that measured LMA using total leaf surface area (e.g., Wang et al. 2019; Schoettle 2009; Springer et al. 2005), as LMA calculated using total leaf surface area are typically 66% lower than LMA calculated using projected leaf area (see Eimil-Fraga et al. 2015 for comparisons). We collected the leaf water potential at turgor loss point (TLP) for 52 species from 24 different papers. We excluded TLP measured on seedlings as TLP is typically 50% lower in seedlings (see Hart et al. 2020 and Samuelson et al. 2019 for a comparison between *Pinus palustris* or Collier & Boyer 1989 for a comparison of *Thuja occidentalis*). Repeated data was removed systematically from the dataset. Hybrids were not included. We attempted to resolve ambiguous names and combined synonyms into a consensus list of conifer species using the Kew World Checklist of Vascular Plant Species (Govaerts et al. 2021) which was cross-referenced with available trait data. Each trait was averaged per species, per publication.

*Comparing the spatial distribution of drought, freeze-thaw cycles, and their combination worldwide*

To estimate the current frequency of freeze-thaw (FT) cycles, we used a global point dataset of maximum and minimum daily temperatures (1975-2018) and long-term daily means (1951-1980) from BerkeleyEarth (http://berkeleyearth.org/data/) with a resolution of ~ 111 km^2^. For every grid cell location, we counted FT days as those whose air temperature range spanned 0⁰C. This cutoff provides a conservative estimate of FT days as it does not capture vegetation freezing due to radiation frosts (Jordan & Smith 1995) or thawing due to daytime radiation when air temperatures remain below zero (Larsen et al 2007). When temperature extremes were missing, we categorized these as possible FT days if the long-term mean was within the 99% confidence interval of long-term means when FT occurs. These confidence intervals were determined separately for each decade to account for warming trends. We then averaged the total number of FT days per year across all years, excluding years with more than 5 days with at least one temperature extreme missing. This produced a dataset where most years are more than 99.95% complete (except 2018 which is 98.21% complete) and 98.18% of grid cells are missing no years. Freeze-thaw point data were interpolated to two rasters using inverse distance weighting and triangulated irregular networking to a resolution of ~ 10km^2^ in QGIS (v3.16, QGIS Development Team 2021, https://qgis.org) and averaged per grid cell.

To estimate potential drought stress from low soil and/or atmospheric water availability we created an index using two variables: minimum monthly soil water content fraction (swc) from the High-Resolution Global Soil-Water Balance data provided by the Consortium for Spatial Information (data covers 1970-2000; CGIAR-CSI; https://cgiarcsi.community/) and monthly vapor pressure deficit (vpd) data from TerraClimate (data covers 1958-2019; https://climatologylab.org; Abatzoglou et al. 2018). To calculate a relative index of potential drought stress, we rescaled data from 1 to 100 (where lower values represented greater soil or atmospheric water availability) and multiplied grid cell values of the two rasters. We then rescaled their products to set the minimum value to 1 (representing the lowest water deficits) and the maximum value to 100 (representing the highest water deficits and stress).

We also created an index to represent the combination of FT stress and potential drought stress (i.e. poly-stress) by rescaling the FT raster data from 1 to 100, then multiplying the FT raster cells by the rescaled drought raster cells. The minimum product of FTi*Di was 3 and the maximum product was 2288. We then rescaled the raster (i.e. the products of FT and Di) to set the minimum value to 1 (representing the lowest combined stress) and the maximum value to 100 (representing the highest combined stress). All raster grid transformations were completed in QGIS using the *raster_normalization* function (Eq. 1). All global land maps were made in R (v4.0, R Core Team, 2021) and QGIS (v3.16, QGIS Development Team 2021, https://qgis.org).

$$Rescaled=\frac{(grid value - MIN grid value) * (MAX scale value-MIN scale value)}{MAX grid value - MIN grid value}+MIN scale value$$

Eq. 1

*Species’ occurrence and trait records in FT-D space*

To find the FT-D space in which species are known to occur, we first obtained occurrence data from GBIF using the *spocc* package (Chamberlain 2018) and the BRAHMS databases of conifer herbarium records (https://herbaria.plants.ox.ac.uk/bol/conifers). We removed duplicate records and thinned occurrence records for each species by removing coordinates that were identical to two decimal places (achieving an approximate maximum point proximity of ~ 1km).We removed geographical outliers using the *CoordinateCleaner* package in R (Zizka et al. 2009) and then assigned FT and D indices based on these coordinates. Coordinates were further cleaned by removing potential outliers in environmental space. We identified potential outliers in environmental space by their isolation forest anomaly score and excluded points with anomaly scores greater than 0.70 (*solitude* package, Srikanth 2020).

To find the FT-D space in which *traits* have been measured, we subjected our compiled trait records to the same process above. We then calculated the percent trait area per total occurrence area.. For illustration, selected five species each from five genera, chosen for their large number and/or wide range of data points, their taxonomic diversity (families Cupressaceae, Pinaceae and Podocarpaceae) and geographic diversity (northern and southern hemisphere). We also list all species, grouped by dominant geographic realm and sorted by their percent trait area per total occurrence area in Supplemental Table S1. We obtained the realm of each datapoint from the Ecoregions 2017 dataset (Dinerstein et al. 2017) and limited each species' number of realms to its three most dominant. For species with more than 10 points, we excluded realms with fewer than 10 points or 5% of the occurrences.

*Conifers and their biomes in worldwide FT-D space*

To identify the worldwide distribution of FT-D space (excluding Antarctica), we first downsampled our FT and D rasters to 1/16 resolution. We then rounded each value to the nearest three index units and tabulated these rounded FT-D combinations. To assign a biome to each location we used the Ecoregions 2017 dataset (Dinerstein et al. 2017) to assign biomes to all conifer occurrence locations. All biomes were represented except “rock and ice” and each species often ended up with multiple biome assignments. We only included biome*species combinations represented by either at least ten points or at least 5% of all biome*species data points. Most notably, this filtering excluded nearly all points in “mangroves” and “flooded grasslands and savannas” biomes. We excluded these biomes because we believed they were miscategorizations and/or of minor importance to our goal.

**Literature Cited**

Abatzoglou, J. T., Dobrowski, S. Z., Parks, S. A., & Hegewisch, K. C. (2018). TerraClimate, a

high-resolution global dataset of monthly climate and climatic water balance from 1958–2015. *Scientific Data, 5*(1), 1-12.

Chamberlain, S. A., & Szöcs, E. (2013). taxize: taxonomic search and retrieval in R.

*F1000Research, 2*.

Chamberlain, S.A. (2018). spocc: Interface to Species Occurrence Data Sources. URL:

https://cran.r-project.org/web/packages/spocc/index.html

Dinerstein, E., Olson, D., Joshi, A., Vynne, C., Burgess, N. D., Wikramanayake, E., ... &

Saleem, M. (2017). An ecoregion-based approach to protecting half the terrestrial realm. *BioScience, 67*(6), 534-545.

Enquist, B. J., Condit, R., Peet, R. K., Schildhauer, M., & Thiers, B. M. (2016).

Cyberinfrastructure for an integrated botanical information network to investigate the ecological impacts of global climate change on plant biodiversity (No. e2615v2). *PeerJ Preprints*.

Farjon, A. (2018). The Kew review: Conifers of the world. Kew bulletin, 73(1), 1-16.

Govaerts, R., Nic Lughadha, E., Black, N. et al. The World Checklist of Vascular Plants, a

continuously updated resource for exploring global plant diversity. Sci Data 8, 215

(2021). https://doi.org/10.1038/s41597-021-00997-6

Jordan, D. N., & Smith, W. K. (1995). Radiation frost susceptibility and the association

between sky exposure and leaf size. *Oecologia, 103*(1), 43-48.

Kattge, J., Diaz, S., Lavorel, S., Prentice, I. C., Leadley, P., Bönisch, G., ... & Wirth, C.

(2011). TRY–a global database of plant traits. *Global Change Biology, 17*(9), 2905-2935.

Larsen, K. S., Ibrom, A., Jonasson, S., Michelsen, A., & Beier, C. (2007). Significance of

cold‐season respiration and photosynthesis in a subarctic heath ecosystem in Northern Sweden. *Global Change Biology, 13*(7), 1498-1508.

R Core Team (2021). R: A language and environment for statistical computing. R Foundation

for Statistical Computing, Vienna, Austria. URL [https://www.R-project.org/](https://www.r-project.org/)

Srikanth, K. (2020). solitude: An Implementation of Isolation Forest. URL:

https://cran.r-project.org/web/packages/solitude/index.html

Zizka, A., Silvestro, D., Andermann, T., Azevedo, J., Duarte Ritter, C., Edler, D., ... &

Antonelli, A. (2019). CoordinateCleaner: Standardized cleaning of occurrence records from biological collection databases. *Methods in Ecology and Evolution, 10*(5), 744-751.

Data references

Bahari et al. 1985. Photosynthesis, water relations, and drought adaptation in six woody species of oak-hickory forests in Central Missouri. Forest Science, 31(3), 557-569.

Baker et al. 2019. Six co-occurring conifer species in northern Idaho exhibit a continuum of hydraulic strategies during an extreme drought year. AoB Plants, 11(5), plz056.

Beikircher & Mayr. 2008. The hydraulic architecture of Juniperus communis L. ssp. communis: shrubs and trees compared. Plant, Cell & Environment, 31(11), 1545-1556.

Beikircher et al. 2010. Restoration of rocky slopes based on planted gabions and use of drought-preconditioned woody species. Ecological Engineering, 36(4), 421-426.

Benavides et al. 2019. The functional trait space of tree species is influenced by the species richness of the canopy and the type of forest. Oikos, 128(10), 1435-1445.

Berry et al. 2015. Vegetation-zonation patterns across a temperate mountain cloud forest ecotone are not explained by variation in hydraulic functioning or water relations. Tree Physiology, 35(9), 925-935.

Bhusal et al. 2021. Evaluation of morphological, physiological, and biochemical traits for assessing drought resistance in eleven tree species. Science of the Total Environment, 779, 146466.

Birmann & Körner. 2009. Nitrogen status of conifer needles at the alpine treeline. Plant Ecology & Diversity, 2(3), 233-241.

Bond et al. 1998. Foliage physiology and biochemistry in response to light gradients in conifers with varying shade tolerance. Oecologia, 120(2), 183-192.

Bouche et al. 2014. A broad survey of hydraulic and mechanical safety in the xylem of conifers. Journal of Experimental Botany, 65(15), 4419-4431.

Bouche et al. 2016. Are needles of Pinus pinaster more vulnerable to xylem embolism than branches? New insights from X-ray computed tomography. Plant, Cell & Environment, 39(4), 860-870.

Bouche et al. 2016. Low intra-tree variability in resistance to embolism in four Pinaceae species. Annals of Forest Science, 73(3), 681-689.

Brodribb & Cochard. 2009. Hydraulic failure defines the recovery and point of death in water-stressed conifers. Plant Physiology, 149(1), 575-584.

Brodribb & Hill. 1999. The importance of xylem constraints in the distribution of conifer species. New Phytologist, 143(2), 365-372.

Brodribb & Hill. 2000. Increases in water potential gradient reduce xylem conductivity in whole plants. Evidence from a low-pressure conductivity method. Plant Physiology, 123(3), 1021-1028.

Brodribb et al. 2010. Xylem function and growth rate interact to determine recovery rates after exposure to extreme water deficit. New Phytologist, 188(2), 533-542.

Burgess et al. 2006. Hydraulic efficiency and safety of branch xylem increases with height in Sequoia sempervirens (D. Don) crowns. Plant, Cell & Environment, 29(2), 229-239.

Carriquí et al. 2020. Cell wall composition strongly influences mesophyll conductance in gymnosperms. The Plant Journal, 103(4), 1372-1385.

Cary & Pittermann. 2018. Small trees, big problems: Comparative leaf function under extreme edaphic stress. American Journal of Botany, 105(1), 50-59.

Cary et al. 2020. Xylem form and function under extreme nutrient limitation: an example from California's pygmy forest. New Phytologist, 226(3), 760-769.

Charra-Vaskou et al. 2012. Drought and frost resistance of trees: a comparison of four species at different sites and altitudes. Annals of Forest Science, 69(3), 325-333.

Charra-Vaskou et al. 2012. Hydraulic efficiency and safety of vascular and non-vascular components in Pinus pinaster leaves. Tree Physiology, 32(9), 1161-1170.

Cherif et al. 2019. Hydraulic traits performances of three pine species in Tunisia. Journal of Agricultural Science, 11(13), 20.

Chin & Sillett. 2019. Within-crown plasticity in leaf traits among the tallest conifers. American Journal of Botany, 106(2), 174-186.

Clute et al. 2021. Hydraulic traits of co‑existing conifers do not correlate with local hydroclimate condition: a case study in the northern Rocky Mountains, U.S.A. Oecologia, 197(2), 1049-1062.

Cochard et al. 2005. Evaluation of a new centrifuge technique for rapid generation of xylem vulnerability curves. Physiologia Plantarum, 124(4), 410-418.

Cochard. 1992. Vulnerability of several conifers to air embolism. Tree Physiology, 11(1), 73-83.

Cochard. 2006. Cavitation in trees. Comptes Rendus Physique, 7(9-10), 1018-1026.

Collier & Boyer. 1989. The water relations of Thuja occidentalis L. from two sites of contrasting moisture availability. Botanical Gazette, 150(4) 445-448.

Condo & Reinhardt. 2019. Large variation in branch and branch-tip hydraulic functional traits in Douglas-fir (Pseudotsuga menziesii) approaching lower treeline. Tree Physiology, 39(8), 1461-1472.

Dai et al. 2020. Frost fatigue and its spring recovery of xylem conduits in ring-porous, diffuse-porous, and coniferous species in situ. Plant Physiology and Biochemistry, 146, 177-186.

de Vasconcellos Barros et al. 2022. Phytogeographical origin determines Tropical Montane Cloud Forest hydraulic trait composition. Functional Ecology, 36(3), 607-621.

Deligöz & Cankara. 2020. Differences in physiological and biochemical responses to summer drought of Pinus nigra subsp. pallasiana and Pinus brutia in a natural mixed stand. Journal of Forestry Research, 31(5), 1479-1487.

DeLucia & Schlesinger. 1991. Resource-use efficiency and drought tolerance in adjacent Great Basin and Sierran plants. Ecology, 72(1), 51-58.

Delzon et al. 2010. Mechanism of water-stress induced cavitation in conifers: bordered pit structure and function support the hypothesis of seal capillary-seeding. Plant, Cell & Environment, 33(12), 2101-2111.

Dietrich et al. 2018. No role for xylem embolism or carbohydrate shortage in temperate trees during the severe 2015 drought. Journal of Ecology, 107(1), 334-349.

Domec & Gartner. 2002. Age- and position-related changes in hydraulic versus mechanical dysfunction of xylem: inferring the design criteria for Douglas-fir wood structure. Tree Physiology, 22(2-3), 91-104.

Domec & Gartner. 2003. Relationship between growth rates and xylem hydraulic characteristics in young, mature and old-growth ponderosa pine trees. Plant, Cell & Environment, 26(3), 471-483.

Ducrey et al. 2008. Variability in growth, carbon isotope composition, leaf gas exchange and hydraulic traits in the eastern Mediterranean cedars Cedrus libani and C. brevifolia. Tree Physiology, 28(5), 689-701.

Dulamsuren et al. 2018. Hydraulic architecture and vulnerability to drought-induced embolism in southern boreal tree species of Inner Asia. Tree Physiology 39(3), 463-473.

Duursma et al. 2005. Estimating leaf-level parameters for ecosystem process models: a study in mixed conifer canopies on complex terrain. Tree Physiology, 25(11), 1347-1359.

Eckert et al. 2020. The maximum carboxylation rate of Rubisco affects CO2 refixation in temperate broadleaved forest trees. Plant Physiology and Biochemistry, 115, 330-337.

Eimil-Fraga et al. 2015. Relationships between needle traits, needle age and site and stand parameters in Pinus pinaster. Trees, 29(4), 1103-1113.

Enright et al. 2014. Stress-tolerator leaf traits determine population dynamics in the endangered New Caledonian conifer Araucaria muelleri. Austral Ecology, 39(1), 60-71.

Enright et al. 2022. The ecology of conifer persistence in tropical rainforests: Podocarpus neriifolius in northern Thailand. Plant Ecology, 223(3), 297-314.

Ewers & Zimmermann. 1984. The hydraulic architecture of balsam fir (Abies balsamea). Physiologia Plantarum, 60(4), 453-458.

Ewers & Zimmermann. 1984. The hydraulic architecture of eastern hemlock (Tsuga canadensis). Canadian Journal of Botany, 62(5), 940-946

Ewers et al. 2000. Influence of nutrient versus water supply on hydraulic architecture and water balance in Pinus taeda. Plant, Cell & Environment, 23(10), 1055-1066.

Fajardo & Siefert. 2016. Temperate rain forest species partition fine-scale gradients in light availability based on their leaf mass per area (LMA). Annals of Botany, 118(7), 1307-1315.

Fan et al. 2022. Functional traits explain growth–mortality trade‑offs in a mixed broadleaf‑conifer forest in northeastern China. European Journal of Forest Research, 141(1), 117-128.

Fellner et al. 2016. Specific leaf area of European Larch (Larix decidua Mill.). Trees, 30, 1237-1244.

Feng et al. 2021. Cavitation fatigue in conifers: a study on eight European species. Plant Physiology, 186(3), 1580-1590.

Field & Brodribb. 2005. A unique mode of parasitism in the conifer coral tree Parasitaxus ustus (Podocarpaceae). Plant, Cell & Environment, 28(10), 1316-1325.

Garcia-Forner et al. 2016. Responses of two semiarid conifer tree species to reduced precipitation and warming reveal new perspectives for stomatal regulation. Plant, Cell & Environment, 39(1), 38-49.

Garnier et al. 2001. Consistency of species ranking based on functional leaf traits. New Phytologist, 152(1), 69-83.

Gonzalez-Benecke et al. 2010. Hydraulic architecture and tracheid allometry in mature Pinus palustris and Pinus elliottii trees. Tree Physiology, 30(3), 361-375.

Gower et al. 1993. Canopy dynamics and aboveground production of five tree species with different leaf longevities. Tree Physiology, 12(4), 327-345.

Grossiord et al. 2016. Precipitation, not air temperature, drives functional responses of trees in semi-arid ecosystems. Journal of Ecology, 105(1), 163-175.

Gyenge et al. 2005. Leaf and whole-plant water relations of the Patagonian conifer Austrocedrus chilensis (D. Don) Pic. Ser. et Bizzarri: implications on its drought resistance capacity. Annals of Forest Science, 62(4), 297-302.

Hacke & Jansen. 2009. Embolism resistance of three boreal conifer species varies with pit structure. New Phytologist, 182(3), 675-686.

Hacke et al. 2000. Influence of soil porosity on water use in Pinus taeda. Oecologia, 124, 495-505.

Hacke et al. 2004. Analysis of circular bordered pit function II. Gymnosperm tracheids with torus-margo pit membranes. American Journal of Botany, 91(3), 386-400.

Hammond et al. 2019. Dead or dying? Quantifying the point of no return from hydraulic failure in drought-induced tree mortality. New Phytologist, 223(4), 1834-1843.

Han et al. 2008. Leaf-age effects on seasonal variability in photosynthetic parameters and its relationships with leaf mass per area and leaf nitrogen concentration within a Pinus densiflora crown. Tree Physiology, 28(4), 551-558.

Han. 2011. Height-related decreases in mesophyll conductance, leaf photosynthesis and compensating adjustments associated with leaf nitrogen concentrations in Pinus densiflora. Tree Physiology, 31(9), 976-984.

Hanley et al. 2021. Relating the climate envelopes of urban tree species to their drought and thermal tolerance. Science of the Total Environment, 753, 142012.

Hikosaka & Shigeno. 2009. The role of Rubisco and cell walls in the interspecific variation in photosynthetic capacity. Oecologia, 160(3), 443-451.

Hikosaka et al. 2021. Intraspecific variations in leaf traits, productivity and resource use efficiencies in the dominant species of subalpine evergreen coniferous and deciduous broad-leaved forests along the altitudinal gradient. Journal of Ecology, 109(4), 1804-1818.

Hoch et al. 2003. Non-structural carbon compounds in temperate forest trees. Plant, Cell & Environment, 26(7), 1067-1081.

Hubbard et al. 2001. Stomatal conductance and photosynthesis vary linearly with plant hydraulic conductance in ponderosa pine. Plant, Cell & Environment, 24(1), 113-121.

Ishii et al. 2014. Pushing the limits to tree height: could foliar water storage compensate for hydraulic constraints in Sequoia sempervirens? Functional Ecology, 28(5), 1087-1093.

Jackson & Spomer. 1979. Biophysical adaptations of four western conifers to habitat water conditions. Botanical Gazette, 140(4), 428-432.

Jager et al. 2015. Soil fertility induces coordinated responses of multiple independent functional traits. Journal of Ecology, 103(2), 374-385.

Jankowski et al. 2017. Cold adaptation drives variability in needle structure and anatomy in Pinus sylvestris L. along a 1,900 km temperate–boreal transect. Functional Ecology, 31(12), 2212-2223.

Jansen et al. 2008. The effect of preparation techniques on SEM-imaging of pit membranes. International Association of Wood Anatomists Journal, 29(2), 161-178.

Jansen et al. 2012. Plasmodesmatal pores in the torus of bordered pit membranes affect cavitation resistance of conifer xylem. Plant, Cell & Environment, 35(6), 1109-1120.

Jin et al. 2019. Conifers but not angiosperms exhibit vulnerability segmentation between leaves and branches in a temperate forest. Tree Physiology, 39(3), 454-462.

Jin et al. 2021. Leaf hydraulic traits of larch and ash trees in response to long-term nitrogen addition in northeastern China. Journal of Plant Ecology, 14(6), 1105-1114.

Johnson et al. 2009. Leaf hydraulic conductance, measured in situ, declines and recovers daily: leaf hydraulics, water potential and stomatal conductance in four temperate and three tropical tree species. Tree Physiology, 29(7), 879-887.

Johnson et al. 2009. Leaf xylem embolism, detected acoustically and by cryo-SEM, corresponds to decreases in leaf hydraulic conductance in four evergreen species. Plant, Cell & Environment, 32(7), 828-836.

Johnson et al. 2011. Hydraulic patterns and safety margins, from stem to stomata, in three eastern US tree species. Tree Physiology, 31(6), 659-668.

Johnson et al. 2016. A test of the hydraulic vulnerability segmentation hypothesis in angiosperm and conifer tree species. Tree Physiology, 36(8), 983-993.

Johnson et al. 2018. Leaf hydraulic parameters are more plastic in species that experience a wider range of leaf water potentials. Functional Ecology, 32(4), 894-903.

Kilgore et al. 2020. Hydraulics of Pinus (subsection Ponderosae) populations across an elevation gradient in the Santa Catalina Mountains of southern Arizona. Madroño, 67(4), 218-226.

Kloeppel et al. 1998. Foliar carbon isotope discrimination in Larix species and sympatric evergreen conifers: a global comparison. Oecologia, 114, 153-159.

Kloeppel et al. 2001. Leaf-level resource use for evergreen and deciduous conifers along a resource availability gradient. Functional Ecology, 14(3), 281-292.

Kunert & Tomaskova. 2020. Leaf turgor loss point at full hydration for 41 native and introduced tree and shrub species from Central Europe. Journal of Plant Ecology, 13(6), 754-756.

Kuusk et al. 2018. Structural controls on photosynthetic capacity through juvenile-to-adult transition and needle ageing in Mediterranean pines. Functional Ecology, 32(6), 1479-1491.

Ladjal et al. 2005. Drought effects on hydraulic conductivity and xylem vulnerability to embolism in diverse species and provenances of Mediterranean cedars. Tree Physiology 25(9), 1109-1117.

Larter et al. 2015. Extreme aridity pushes trees to their physical limits. Plant Physiology, 168(3), 804-807.

Larter et al. 2017. Aridity drove the evolution of extreme embolism resistance and the radiation of conifer genus Callitris. New Phytologist, 215(1), 97-112.

Laughlin et al. 2011. Climatic constraints on trait-based forest assembly. Journal of Ecology, 99(6), 1489-1499.

Laughlin et al. 2020. Climatic limits of temperate rainforest tree species are explained by xylem embolism resistance among angiosperms but not among conifers. New Phytologist, 226(3), 727-740.

Li et al. 2008. Evaluation of centrifugal methods for measuring xylem cavitation in conifers, diffuse- and ring-porous angiosperms. New Phytologist, 177(2), 558-568.

Linton et al. 1998. Limits to water transport in Juniperus osteosperma and Pinus edulis: implications for drought tolerance and regulation of transpiration. Functional Ecology, 12(6), 906-911.

Liu et al. 2020. Variations in leaf economics spectrum traits for an evergreen coniferous species: Tree size dominates over environment factors. Functional Ecology, 34(2), 458-467.

Lopez et al. 2013. Vulnerability to cavitation, hydraulic efficiency, growth and survival in an insular pine (Pinus canariensis). Annals of Botany, 111(6), 1167-1179.

Lu et al. 1996. Water relations of adult Norway spruce (Picea abies L.) Karst) under soil drought in the Vosges mountains: Whole-tree hydraulic conductance, xylem embolism and water loss regulation. Annals of Forest Science, 53(1), 113-121.

Lusk et al. 2003. Photosynthetic differences contribute to competitive advantage of evergreen angiosperm trees over evergreen conifers in productive habitats. New Phytologist, 160(2), 329-336.

Lusk. 2001. Leaf life spans of some conifers of the temperate forests of South America. Revista Chilena de Historia Natural, 74(3), 711-718.

Maherali & DeLucia. 2000. Xylem conductivity and vulnerability to cavitation of ponderosa pine growing in contrasting climates. Tree Physiology, 20(13), 859-867.

Maherali et al. 2006. Functional coordination between leaf gas exchange and vulnerability to xylem cavitation in temperate forest trees. Plant, Cell & Environment, 29(4), 571-583.

Mantova et al. 2021. The interplay of hydraulic failure and cell vitality explains tree capacity to recover from drought. Physiologia Plantarum, 172(1), 247-257.

Martinez-Vilalta & Piñol. 2002. Drought-induced mortality and hydraulic architecture in pine populations of the NE Iberian Peninsula. Forest Ecology and Management, 161(1-3), 247-256.

Martinez-Vilalta et al. 2009. Hydraulic adjustment of Scots pine across Europe. New Phytologist, 184(2), 353-364.

Mayr & Sperry. 2010. Freeze–thaw-induced embolism in Pinus contorta: centrifuge experiments validate the ‘thaw-expansion hypothesis’ but conflict with ultrasonic emission data. New Phytologist, 185(4), 1016-1024.

Mayr et al. 2002. Winter-drought induced embolism in Norway spruce (Picea abies) at the Alpine timberline. Physiologia Plantarum, 115(1), 74-80.

Mayr et al. 2003. Repeated freeze-thaw cycles induce embolism in drought stressed conifers (Norway spruce, stone pine). Planta, 217(3), 436-441.

Mayr et al. 2003. Winter at the alpine timberline. Why does embolism occur in Norway Spruce but not in Stone Pine? Plant Physiology, 131(2), 780-792.

Mayr et al. 2003. Winter-embolism in a krummholz shrub (Pinus mugo) growing at the alpine timberline. Austrian Journal of Forest Science, 120(1), 29-38.

Mayr et al. 2006. Frost drought in conifers at the alpine timberline: xylem dysfunction and adaptations. Ecology, 87(12), 3175-3185.

McCulloh et al. 2014. The dynamic pipeline: Hydraulic capacitance and xylem hydraulic safety in four tall conifer species. Plant, Cell & Environment, 37(5), 1171-1183.

McElrone et al. 2004. Variation in xylem structure and function in stems and roots of trees to 20 m depth. New Phytologist, 163(3), 507-517.

McIntire et al. 2022. Allometric relationships for predicting aboveground biomass, sapwood, and leaf area of two-needle piñon pine (Pinus edulis) amid open-grown conditions in central New Mexico. Forest Science, 68(2), 152-161.

Mediavilla & Escudero. 2003. Relative growth rate of leaf biomass and leaf nitrogen content in several mediterranean woody species. Plant Ecology, 168(2), 321-332.

Mediavilla et al. 2014. Ontogenetic transition in leaf traits: a new cost associated with the increase in leaf longevity. Journal of Plant Ecology, 7(6), 567-575.

Mitchell. 1998. Acclimation of Pacific yew (Taxus brevifolia) foliage to sun and shade. Tree Physiology, 18(11), 749-757.

Mitchell. 2001. Growth limitations for conifer regeneration under alternative silvicultural systems in a coastal montane forest in British Columbia, Canada. Forest Ecology and Management, 145(1-2), 129-136.

Morcillo et al. 2022. Post-drought conditions and hydraulic dysfunction determine tree resilience and mortality across Mediterranean Aleppo pine (Pinus halepensis) populations after an extreme drought event. Tree Physiology, 42(7), 1364-1376.

Nagano et al. 2009. Needle traits of an evergreen, coniferous shrub growing at wind‐exposed and protected sites in a mountain region: does Pinus pumila produce needles with greater mass per area under wind‐stress conditions? Plant Biology, 11, 94-100.

Ning et al. 2022. Differences in growth pattern and response to climate warming between Larix olgensis and Pinus koraiensis in Northeast China are related to their distinctions in xylem hydraulics. Agricultural and Forest Meteorology, 312, 108724.

Ogle et al. 2009. Hierarchical statistical modeling of xylem vulnerability to cavitation. New Phytologist, 182(2), 541-554.

Olano et al. 2017. Sex determines xylem anatomy in a dioecious conifer: hydraulic consequences in a drier world. Tree Physiology, 37(11), 1493-1502.

Oliveras et al. 2003. Hydraulic properties of Pinus halepensis, Pinus pinea and Tetraclinis articulata in a dune ecosystem of Eastern Spain. Plant Ecology, 169, 131-141 .

Olson et al. 2018. Plant height and hydraulic vulnerability to drought and cold. Proceedings of the National Academy of Sciences, 115(29), 7551-7556.

Onoda et al. 2017. Physiological and structural tradeoffs underlying the leaf economics spectrum. New Phytologist, 214(4), 1447-1463.

Peng et al. 2012. The age-related changes of leaf structure and biochemistry in juvenile and mature subalpine fir trees (Abies faxoniana Rehder & E.H. Wilson) along an altitudinal gradient. Polish Journal of Ecology, 60(2), 311-321.

Pinol & Sala. 2000. Ecological implications of xylem cavitation for several Pinaceae in the Pacific Northern USA. Functional Ecology, 14(5), 538-545.

Pittermann & Sperry. 2006. Analysis of freeze-thaw embolism in conifers. The interaction between cavitation pressure and tracheid size. Plant Physiology, 140(1), 374-382.

Pittermann et al. 2006. Inter-tracheid pitting and the hydraulic efficiency of conifer wood: the role of tracheid allometry and cavitation protection. American Journal of Botany, 93(9), 1265-1273.

Pittermann et al. 2010. The relationships between xylem safety and hydraulic efficiency in the Cupressaceae: the evolution of pit membrane form and function. Plant Physiology, 53(4), 1919-1931.

Pittermann et al. 2012. Cenozoic climate change shaped the evolutionary ecophysiology of the Cupressaceae conifers. Proceedings of the National Academy of Sciences, 109(24), 9647-9652.

Pleijel et al. 2021. Mercury accumulation in leaves of different plant types – the significance of tissue age and specific leaf area. Biogeosciences, 18(23), 6313-6328.

Pockman & Sperry. 2000. Vulnerability to xylem cavitation and the distribution of Sonoran Desert vegetation. American Journal of Botany, 87(9), 1287-1299.

Poorter et al. 2000. Leaf optical properties in Venezuelan cloud forest trees. Tree Physiology, 20(8), 519-526.

Poulos & Berlyn. 2007. Variability in Needle Morphology and Water Status of Pinus cembroides across an Elevational Gradient in the Davis Mountains of West Texas, USA. The Journal of the Torrey Botanical Society, 134(2), 281-288.

Prendin et al. 2018. Xylem anatomical adjustments prioritize hydraulic efficiency over safety as Norway spruce trees grow taller. Tree Physiology, 38(8), 1088-1097.

Qin & Shangguan. 2019. Effects of forest types on leaf functional traits and their interrelationships of Pinus massoniana coniferous and broad-leaved mixed forests in the subtropical mountain, Southeastern China. Ecology and Evolution, 9(12), 6922-6932.

Read. 1985. Photosynthetic and growth responses to different light regimes of the major canopy species of Tasmanian cool temperate rainforest. Austral Ecology, 10(3), 327-334.

Reich et al. 1998. Relationships of leaf dark respiration to leaf nitrogen, specific leaf area and leaf life-span: a test across biomes and functional groups. Oecologia, 114(4), 471-482.

Rosas et al. 2021. Are leaf, stem and hydraulic traits good predictors of individual tree growth? Functional Ecology, 35(11), 2435-2447.

Royer et al. 2010. Leaf economic traits from fossils support a weedy habit for early angiosperms. American Journal of Botany, 97(3), 438-445.

Saenz-Romero et al. 2013. Genetic variation of drought-induced cavitation resistance among Pinus hartwegii populations from an altitudinal gradient. Acta Physiologiae Plantarum, 35(10), 2905-2913.

Saenz-Romero et al. 2017. Mexican conifers differ in their capacity to face climate change. Journal of Plant Hydraulics, 4, e-003.

Samuelson et al. 2019. Drought tolerance of a Pinus palustris plantation. Forest Ecology and Management, 451, 117557 .

Schmiege et al. 2021. Contrasting physiological traits of shade tolerance in Pinus and Podocarpaceae native to a tropical Vietnamese forest: insight from an aberrant flat-leaved pine. Tree Physiology, 41(2), 223-239.

Schmiege et al. 2021. Respiratory temperature responses of tropical conifers differ with leaf morphology. Functional Ecology, 35(7), 1408-1423.

Schoonmaker et al. 2010. Hydraulic acclimation to shading in boreal conifers of varying shade tolerance. Plant, Cell & Environment, 33(3), 382-393.

Schulte et al. 1992. Pressure–volume analysis of tissue water relations parameters for individual fascicles of loblolly pine (Pinus taeda L.). Tree Physiology, 10(4), 381-389.

Singh et al. 2006. Patterns in water relations of central Himalayan trees. Tropical Ecology, 47(2), 159-182.

Soh et al. 2017. Palaeo leaf economics reveal a shift in ecosystem function associated with the end-Triassic mass extinction event. Nature Plants, 3, 17104.

Sperry & Ikeda. 1997. Xylem cavitation in roots and stems of Douglas-fir and white fir. Tree Physiology, 17(4), 275-280.

Sperry & Sullivan. 1992. Xylem embolism in response to freeze-thaw cycles and water stress in ring-porous, diffuse-porous, and conifer species. Plant Physiology, 100(2), 605-613.

Sperry & Tyree. 1990. Water-stress-induced embolism in three species of conifers. Plant, Cell & Environment, 13(5), 427-346.

Stout & Sala. 2003. Xylem vulnerability to cavitation in Pseudotsuga menziesii and Pinus ponderosa from contrasting habitats. Tree Physiology, 23(1), 43-50.

Strahan et al. 2016. Shifts in community-level traits and functional diversity in a mixed conifer forest: a legacy of land-use change. Journal of Applied Ecology, 53(6), 1755-1765.

Tian et al. 2016. Leaf morphological and anatomical traits from tropical to temperate coniferous forests: Mechanisms and influencing factors. Scientific Reports, 6(1), 1-10.

Tissue et al. 2005. Stomatal and non-stomatal limitations to photosynthesis in four tree species in a temperate rainforest dominated by Dacrydium cupressinum in New Zealand. Tree Physiology, 25(4), 447-456.

Tognetti et al. 2001. Stem hydraulic properties and xylem vulnerability to embolism in three co-occurring Mediterranean shrubs at a natural CO2 spring. Australian Journal of Plant Physiology, 28(4), 257-268.

Turner et al. 2000. Tree leaf form in Brunei: a heath forest and a mixed dipterocarp forest compared. Biotropica, 32(1), 53-61.

Tyree & Dixon. 1986. Water stress induced cavitation and embolism in some woody plants. Physiologia Plantarum, 66(3), 397-405.

Tyree et al. 1998. Vulnerability to drought-induced embolism of Bornean heath and dipterocarp forest trees. Tree Physiology, 18(8-9), 583-588.

Urrutia-Jalabert et al. 2018. Elucidating the hydraulic vulnerability of the longest-lived Southern Hemisphere conifer to aridification. Forest Ecology and Management, 430, 472-484.

Vander Willigen et al. 2000. Xylem hydraulic characteristics of subtropical trees from contrasting habitats grown under identical environmental conditions. New Phytologist, 145(1), 51-59.

Walters & Gerlach. 2013. Intraspecific growth and functional leaf trait responses to natural soil resource gradients for conifer species with contrasting leaf habit. Tree Physiology, 33(3), 297-310.

Wang et al. 2014. Leaf trait variation captures climate differences but differs with species irrespective of functional group. Journal of Plant Ecology, 8(1), 61-69.

Wang et al. 2018. Enhanced cell dehydration tolerance and photosystem stability facilitate the occupation of cold alpine habitats by a homoploid hybrid species, Picea purpurea. AoB Plants, 10(5), ply053.

Warren et al. 2021. Divergent species-specific impacts of whole ecosystem warming and elevated CO2 on vegetation water relations in an ombrotrophic peatland. Global Change Biology, 27(9), 1820-1835.

Wei et al. 2022. Genetic variation in leaf traits and gas exchange responses to vapour pressure deficit in contrasting conifer species. Functional Ecology, 36(4), 1036-1046.

Willson & Jackson. 2006. Xylem cavitation caused by drought and freezing stress in four co-occurring Juniperus species. Physiologia Plantarum, 127(3), 374-382.

Willson et al. 2008. Hydraulic traits are influenced by phylogenetic history in the drought-resistant, invasive genus Juniperus (Cupressaceae). American Journal of Botany, 95(3), 229-314.

Wyka et al. 2012. Responses of leaf structure and photosynthetic properties to intra-canopy light gradients: a common garden test with four broadleaf deciduous angiosperm and seven evergreen conifer tree species. Oecologia, 170(1), 11-24.

Yan et al. 2012. Needle-age related variability in nitrogen, mobile carbohydrates, and δ13C within Pinus koraiensis tree crowns. PLOS One, 7(4), e35076.

Zhao et al. 2008. Altitudinal differences in the leaf fitness of juvenile and mature alpine spruce trees (Picea crassifolia). Tree Physiology, 28(1), 133-141.
